# O_2_-Tuned Protein Synthesis Machinery in *Escherichia coli*-Based Cell-Free System

**DOI:** 10.3389/fbioe.2020.00312

**Published:** 2020-04-09

**Authors:** Xiaomei Lin, Caijin Zhou, Songbiao Zhu, Haiteng Deng, Jisong Zhang, Yuan Lu

**Affiliations:** ^1^Key Laboratory of Industrial Biocatalysis, Ministry of Education, Department of Chemical Engineering, Tsinghua University, Beijing, China; ^2^The State Key Lab of Chemical Engineering, Department of Chemical Engineering, Tsinghua University, Beijing, China; ^3^Ministry of Education Key Laboratory of Bioinformatics, Center for Synthetic and Systematic Biology, School of Life Sciences, Tsinghua University, Beijing, China

**Keywords:** cell-free system, tube-in-tube reactor, oxygen-tuned protein synthesis, ATP analysis, reactive oxygen species (ROS), proteomic analysis

## Abstract

Involved in most aerobic biochemical processes, oxygen affects cellular functions, and organism behaviors. Protein synthesis, as the underlying biological process, is unavoidably affected by the regulation of oxygen delivery and utilization. Bypassing the cell wall, cell-free protein synthesis (CFPS) systems are well adopted for the precise oxygen regulation analysis of bioprocesses. Here a reliable flow platform was developed for measuring and analyzing the oxygen regulation on the protein synthesis processes by combining *Escherichia coli*-based CFPS systems and a tube-in-tube reactor. This platform allows protein synthesis reactions conducted in precisely controlled oxygen concentrations. For analysis of the intrinsic role of oxygen in protein synthesis, O_2_-tuned CFPS systems were explored with transcription-translation related parameters (transcripts, energy, reactive oxygen species, and proteomic pathway analysis). It was found that 2% of oxygen was the minimum requirement for protein synthesis. There was translation-related protein degradation in the high oxygen condition leading to a reduction. By combining the precise gas level controlling and open biosystems, this platform is also potential for fundamental understanding and clinical applications by diverse gas regulation in biological processes.

## Introduction

Oxygen, serving as essential nutrition of living life, is the critical substrate of many physiological processes in cells, such as ATP generation in aerobic metabolism, which is considered as the energy supply of thousands of biochemical reactions as well as many oxidative reactions (Lindahl, 2008; Heffner et al., 2013; Palmer and Clegg, 2014). Each life is well adopted to a particular oxygen concentration so that changes in oxygen concentration will cause a series of complicated adaptive efforts or misfunction on cells, tissues, and organisms (Ivan et al., 2001; Jaakkola et al., 2001). Adaptions are including but not limited to enzyme activity, membrane transport (Moore et al., 2018), proliferation (Carmeliet and Jain, 2000; Ke and Costa, 2006; Mohyeldin et al., 2010; Heffner et al., 2013), and systemic inflammatory response. After a long period of evolution, the atmospheric conditions were transferred into the present stable element’s composition, the 21% oxygen concentration (Heffner et al., 2013). However, the natural atmospheric condition does not actually reflect the oxygen microenvironments typically in cellular pathways, and many physiological reactions are conducted in low-level oxygen tension, generally in 2–9% (Brahimi-Horn and Pouysségur, 2007; Mohyeldin et al., 2010).

Although oxygen is vital for life, the mechanisms of oxygen sensing and oxygen utilization in cells are still not completely understood, for the limitation of precise control on gradient oxygen concentrations and effective methods to quantify oxygen concentration in its physiological microenvironment (Roussakis et al., 2015). For gas controlling, pioneering efforts in microfluidic devices have demonstrated that it is possible to conduct oxygen concentrations in tiny gradients by physical diffusion equilibrium, biological cellular uptake, and chemical generation for the study of bacterium growth, cell differentiation, migration and disease mechanism (Leclerc et al., 2004; Lam et al., 2009; Adler et al., 2010; Abaci et al., 2012). Moreover, the ineffective and destructive oxygen concentration quantifying methods still limit the fundamental physiological oxygen-derived research (Roussakis et al., 2015). Clark-style electrodes (Clark et al., 1953; Nozue et al., 1997; Buerk, 2004) and luminescent optical sensors (Sud et al., 2006; Wang and Hu, 2012) are commonly used for oxygen measurement in biological process research, which is either focusing on the extracellular measurement or intracellular dye toxicity (Brennan et al., 2014). Therefore, constructing a more flexible gas controlling and measuring systems are critical challenges for the research of essential oxygen affecting cellular processes.

Cell-free synthetic biology, an emerging biological tool for the study of biological reactions without using the intact cells, has been widely used for the fundamental research in biomolecules, pathways, and whole-cell interactions (Perez et al., 2016). With the advantage of bypassing cell membranes, cell-free systems provide unprecedented freedom for applications of precise physiological analysis researches (Shin and Noireaux, 2012; Lu, 2017; Marshall et al., 2018). Therefore, cell-free systems are well-suitable for oxygen-tuned biological change analysis that could directly reflect the intracellular response to the microenvironment changes. Recently, the tube-in-tube reactor, as a powerful tool for studying the gas-liquid system, has shown significant advantages, which was widely used in gas-liquid reactions in flow mode (Koos et al., 2011; O’brien et al., 2011; Polyzos et al., 2011; Brzozowski et al., 2015). As the device’s name suggests, the tube-in-tube reactor consists of a semipermeable inner tubing and a gas non-permeable outer tubing (Yang and Jensen, 2013; Zhang et al., 2017). Due to the high gas permeability of the inner tubing, the tube-in-tube reactor has a rapid oxygen diffusion rate, which allows oxygen to penetrate rapidly into the liquid in about 30 s (Zhang et al., 2017, 2019). In particular, by using the tube-in-tube reactor, it can easily realize the accurate control and alteration of oxygen concentration in liquid by changing the volume fraction of oxygen at the oxygen-mediated reaction process (Petersen et al., 2012; Ringborg et al., 2017; Burkholder et al., 2019).

Here, a new robust method for oxygen-tuned biological regulation was presented by combining the tube-in-tube flow reactor technology and cell-free systems, in which a physiological process in a precise oxygen concentration was conducted. By exposing cell-free protein synthesis (CFPS) systems to 0–100% oxygen gradients, the features of oxygen regulation in fluorescent protein sfGFP (super fold green fluorescent protein) synthesis were analyzed among energy, transcription, translation, and pathway networks.

## Materials and Methods

### Tube-in-Tube Device

The tube-in-tube reactor was composed of an inner semipermeable membrane tube and an outer impermeable tube. The inner tube was fabricated by the materials of Teflon AF-2400 with an inner diameter of 0.6 mm, and the outer tube was made by polytetrafluoroethylene (PTFE) with an inner diameter of 3.175 mm. Due to the high permeability and short mass transfer distance (0.6 mm) of the inner tube, oxygen could penetrate rapidly into the reaction substrates and saturated the reaction substrates in 30 s (Zhang et al., 2017). A total of 300 μL cell-free reactants were injected into the inner tube through a Harvard pump (Harvard, United States). Twenty microliters of reactants were pushed out and gathered for further analysis at every 20 min in 2 h (Supplementary Figures S1, S2). More details were shown in the Supplementary Material.

### Cell Extract Preparation

For standard cell-free expression reactions, *E. coli* Rosetta (DE3) were fermented in 4 L of 2xYTP (1.6% tryptone; 1% yeast extraction; 0.5% NaCl; 40 mM K_2_HPO_4_; 22 mM KH_2_PO_4_) containing chloramphenicol (34 μg/mL) at 37°C, 300 rpm. Cells were harvested in the late logarithmic growth phase (∼4 h, OD600 = 2). Cell pellets were washed with ice-cold S30A buffer (14 mM magnesium glutamate; 60 mM potassium glutamate; 50 mM Tris, pH 7.7) three times. Cells were resuspended in S30A buffer (1 mL buffer for 1 g of wet cells) for disruption in a high-pressure homogenizer (1,000 bar). Then lysate was centrifuged at 12,000 × g for 10 min at 4°C. The supernatant was incubated at 37°C for 80 min after 3 mM DTT added and centrifuged at 12,000 × g for 10 min at 4°C again. Then it was dialyzed in molecular porous membrane tubing (6-8 KD MWCO) for 3h at 4°C with magnetic stirring. The dialysate was then centrifuged at 12,000 × g for 10 min at 4°C, flash frozen and stored at −80°C (Sun et al., 2013; Supplementary Figure S3).

### Cell-Free Reaction

Cell-free reagents for tube-in-tube reactions were assembled on ice as generally described and immediately injected into the inner tube for incubation at 37°C. The general cell-free reaction mixture consisted of the following components: 30% S30 cell extract (v/v%); 15–20 ng/μL DNA templates; 175 mM potassium glutamate; 10 mM ammonium glutamate; 2.7 mM potassium oxalate monohydrate; 10 mM magnesium glutamate; 50 mM each of 19 amino acids without glutamic acid; 3 mM phosphenol pyruvate (PEP); 1 mM putrescine; 1.5 mM spermidine; 0.33 mM nicotinamide adenine dinucleotide (NAD); 1.2 mM ATP; 0.86 mM each of CTP, GTP and UTP; 0.27 mM coenzyme A; 170 μg/mL tRNA; 34 μg/mL folinic; T7 RNA polymerase prepared from *E. coli* BL21 (DE3) cell extract; reactions also contained 2% PEG8000 (Sun et al., 2013; Supplementary Figure S4).

### Fluorescence Measurement and Data Processing

For fluorescent protein sfGFP measurement, the cell-free samples were diluted and measured in the plate reader (TECAN infinite M200 Pro) with the wavelength of excitation at 485 nm and emission at 520 nm. To minimize the error caused by different batches of cell extracts or other reagents, the fluorescence values were normalized.

Since the long-term storage of cell-free reagents would lead to the fluctuation in fluorescence production, one of the time points (120 min) of 10% treatment was used for the normalization of each cell-free reactions from the same batch of cell-free reagents. The fluorescence of 120 min time point of 10% (*F*_106_) was considered as the same value at each experiment, that the other fluorescence (*F*_*X*_) was calculated as follow: *F*_*XN*_ = *F*_*X*_/*F*_106_^∗^0.825 (0.825, the relative fluorescence to one of 21% treatment in the time point of 150 min). Experiments were run more than twice.

### The ROS Level Analysis

The tube-in-tube cell-free reaction samples were determined by Reactive Oxygen Species Assay Kit (Beyotime, S0033) through the DCFH-DA ROS probe. Cell-free reactions without sfGFP expression were injected into the tube-in-tube reactor along with the DCFH-DA probe for incubation directly. At predetermined time intervals, samples were removed onto the ice. Measure the fluorescence intensity in the plate reader (TECAN infinite M200 Pro) with the wavelength of excitation at 485 nm and emission at 520 nm.

### The ATP Level Analysis

The ATP concentrations were determined by ATP Assay Kit (Beyotime, S0026) through firefly luciferase. Cell-free samples were diluted into lysis buffer and added to the diluted working solution. Luminescence was determined by the plate reader (TECAN infinite M200 Pro). Series concentrations of ATP were used for a standard curve to calculate the ATP concentration of samples.

### RNA Extraction and Target mRNA Quantification

The total mRNA was extracted from cell-free samples by Eastep Super Total RNA Extraction Kit. Then it was stored at −80°C, or the reverse transcription was performed immediately by FastKing RT Kit (With gDNase) (TIANGEN, KR116). The cDNAs were quantified by TranStart Green qPCR SuperMix kit (TranStart, AQ101). The PCR product of sfGFP amplified by the same primers as qPCR was used for the standard curve to relate the sfGFP cDNA concentrations with CT values read by ABI 7300 Real-Time PCR system.

### HPLC-MS/MS Analysis

The samples were separated by SDS-PAGE (12% separating gel) for further MS analysis. The proteins were analyzed by Center of Biomedical Analysis, Tsinghua University. The peptide samples were injected to Q-Exactive mass spectrometor for analysis after In-gel digestion process. The raw files were processed by using the MaxQuant software version 1.6.0.16 (Max Planck Institute, Munich, Germany). The *Escherichia coli* database (strain K12, UniProt release 17/03/2019; containing 29,810 entries) was used by the search engine andromeda implemented in MaxQuant. The criteria for the searching process were listed below: full tryptic specificity required, two missed cleavage tolerance, 20 ppm and 0.02 Da for precursor and fragment ion mass tolerance, respectively. The variable modifications were oxidation on methionine and acetylation on protein N-terminal, and the fixed modifications were carbamidomethylation on cysteine. Both peptide spectrum matches (PSMs) and protein identifications were filtered based on 1% false discovery rate (FDR) based on decoy search for revert mode. The relative label-free quantification (LFQ) of proteomics data based on precursor (MS1) signal intensities were used for quantifying proteins across biological samples. The algorithm MaxLFQ implemented in MaxQuant software was used for LFQ calculation.

One-way ANOVA method and Turkey *post-hoc* analysis were used to determine the significant difference among groups with different oxygen concentrations. *P*-value below 0.05 was considered to be significant. Gene ontology (GO) enrichment analysis was performed using DAVID tools (version 6.8, The Database for Annotation, Visualization and Integrated Discovery) with the complete *Escherichia coli* genome information as the background (Jiao et al., 2012). Visualization of results, including the GO annotations and volcano, were conducted with R packages GOplot, ggplot2, in RStudio (version 1.1, base R 3.5).

## Results and Discussion

### Design of Oxygen Gradient Generator

**FIGURE 1:**
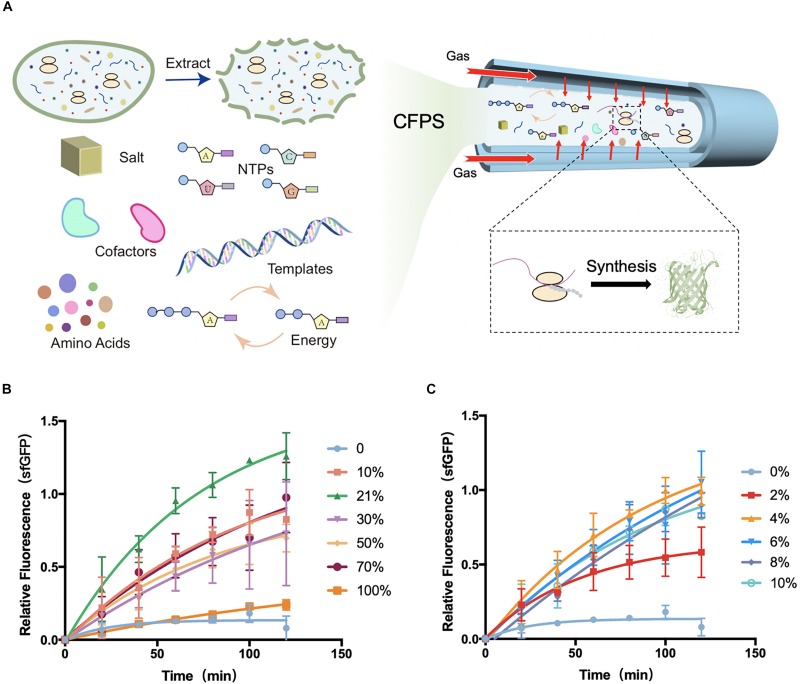
Schematic diagram of tube-in-tube devices for cell-free protein synthesis and fluorescence gained from devices. **(A)** Cell-free protein synthesis (CFPS) system was injected into the inner tube of the tube-in-tube reactor, surrounded by the controllable gas. **(B)** Normalized fluorescence of sfGFP synthesized in cell-free systems in the tube-in-tube reactor oxygen tensions of 0, 10, 21, 30, 50, 70, and 100% in 2 h and 37°C. **(C)** Normalized fluorescence of sfGFP synthesized in cell-free systems in the tube-in-tube reactor with oxygen tensions of 0, 2, 4, 6, 8, and 10% in 2 h and 37°C. Values were calculated as the mean of biological replicates.

The fabrication of the tube-in-tube reactor was constructed with a semipermeable fluoropolymer inner tube (Teflon AF-2400) encased by a gas-non-permeable outer tube (Figure 1A). The Teflon AF-2400 inner tubing is highly permeable for gas but non-permeable for liquid with an inner diameter of 0.6 mm and an outer diameter of 0.8 mm. The structure of the reactor has been introduced in detail previously (Yang and Jensen, 2013; Zhang et al., 2017, 2019; Zhou et al., 2020). The cell-free reactions were injected into the inner tube with a Harvard pump (Harvard, United States). The impermeable outer tube was made of PTFE with an inner diameter of 1.6 mm and an outer diameter of 3.175 mm. Different tensions of oxygen were created by an oxygen stream and a nitrogen stream whose flow rates were controlled by two thermal mass flow controllers (Sevenstar, Beijing, China), respectively (Supplementary Figure S1).

Because of the rapid oxygen diffusion rate (30 s) from the gas phase into the reaction substances in the tube-in-tube reactor, the reaction substrates in the inner tube were always fully saturated with oxygen throughout the protein synthesis process (several hours). For calculating the saturated oxygen concentration of reaction substrates, Henry’s law was used as following:

$$C^{*}=K\,y$$

Where *C*^∗^ (mg/L) is the saturated oxygen concentration of reaction substrates, *y* is the gas volume fraction of oxygen in mixture gas, and *K* [mg/(L)] is Henry’s constant with a constant value of 34.4505 mg/(L) at 37°C. With this formula, the oxygen concentrations in cell-free liquid could be calculated, and the saturation concentrations of oxygen at different oxygen tensions were shown in Supplementary Figure S5.

### Cell-Free Protein Synthesis in Precise Gradients of Oxygen

For the global analysis of the influence of oxygen concentration in CFPS, CFPS reactions for sfGFP (Supplementary Figure S6) in different levels of oxygen were firstly conducted in the tube-in-tube reactor. In this analysis platform, cell-free reaction systems were injected into the inner tube of the tube-in-tube reactor for protein synthesis. The gas tensions varied from 0 to 100% were continuously delivered by changing the flow rates of nitrogen and oxygen streams and maintaining the overall flow rate at 30 mL/min. Before performing the protein synthesis in the tube-in-tube reactor, the cell-free reactants were placed on the ice to stop the synthesis, ensuring that the reactions were performed at a certain oxygen condition.

The protein synthesis features were first explored at a range of O_2_ tension varied from 0 to 100% to investigate the effect of oxygen concentration in CFPS. The model *E. coli*-based cell-free system was employed for this investigation. All of the cell-free reactions for oxygen concentration investigation were performed at 37°C as the natural growth environment of *E. coli*. Experiments with oxygen tensions of 0, 10, 21, 30, 50, 70, and 100% were carried out to separately analyze the protein synthesis characteristics (Figure 1B and Supplementary Figure S7A). To minimize the error from the reagents, the fluorescence was normalized among each batch experiment. Partial fluorescence data were shown in the Supplementary Material (Supplementary Figure S8). As shown in Figure 1B, the natural atmosphere condition 21% oxygen showed the highest yields and protein synthesis rate among the conditions. With the increasing of the oxygen concentration, the protein synthesis rate increased obviously between the tension of 10 and 70%, but a drastic decline in the 100%. As expected, the synthesis was greatly limited when there was no oxygen (0% oxygen and 100% nitrogen) delivery into the synthesis reactions, suggesting that the oxygen was an essential substrate or intermediate in the protein synthesis. As the fluorescence of green fluorescent protein depended on the oxygen oxidation, the expression of 0% oxygen treatment was confirmed with fluorescent recovery (Supplementary Figure S9). As schematically illustrated in Figure 1B, although the synthesis was greatly limited without oxygen, certain low oxygen (10%) was required for the completely functional protein synthesis. However, the high-level oxygen showed the inhibition of protein synthesis in the cell-free system.

Based on the above investigation, the exact oxygen (0–10%) were further investigated for protein synthesis to seek the minimum oxygen concentration. Experiments were performed at the same conditions except for the gradient of oxygen tension, which was ranging from 0 to 10% (Figure 1C and Supplementary Figure S7B). As before, there was little protein synthesis in 0% oxygen condition, while an increasing protein yield appeared at 2%. Until at 4% oxygen, the protein yields were recovered to the high level, suggesting that only a small amount of oxygen (higher than 2%) was required for protein synthesis in *E. coli*-based cell-free systems. This phenomenon of low oxygen concentration requirement of protein synthesis is consistent with previous literature focusing on the *in vivo* systems. Earlier study had found that as little as 0.5% of oxygen could lead to a slow but significant recovered cell growth with protein synthesis in *E. coli*, and a full recovery was at the oxygen tension of ∼7%. However, the condition of 0% oxygen showed a stopping division and protein synthesis (Polinkovsky et al., 2009). From the above investigation and discussion, the tube-in-tube reactor with the excellent gas controlling feature showed great potential for the fundamental investigation of the effect of oxygen concentration on biological processes in the microscale environment with the cell-free system. Moreover, this flexible CFPS device potentially can be applied to different protein synthesis requirements for tunable synthesis rate regulated by the oxygen concentration.

In general, there were several essential observations in the above section. (i) At the 0% oxygen condition, the protein synthesis was limited. (ii) A small amount (larger than 2%) of oxygen was sufficient for the complete function of protein synthesis, and there was a maximum yield in 21% oxygen. (iii) The yield of protein synthesis was not highly dependent on the oxygen at a range of 4–70%, but a substantial decline at 100%. These findings pose a question of what oxygen affects the process of protein synthesis. There might be some changes in the oxygen metabolic pathway or the direct effects on the transcriptional and translational processes.

### Transcription in O_2_-Tuned Cell-Free Systems

**FIGURE 2:**
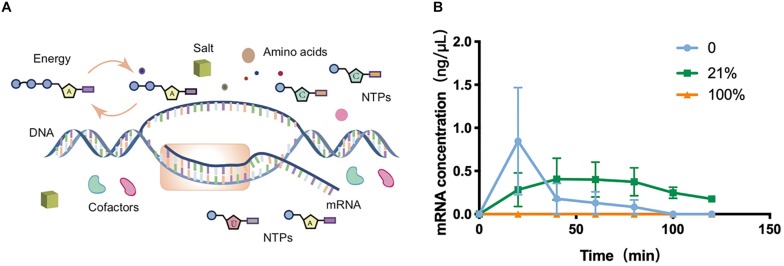
Transcription in cell-free systems. **(A)** Schematic diagram of the transcription process in the cell-free protein synthesis system. **(B)** Transcriptional mRNA analysis of samples incubated in 0, 21, and 100%. Values were calculated as the mean of biological replicates.

To gain a more systematic insight into the effect of oxygen concentration on CFPS processes, the transcriptional level was next quantitatively characterized among O_2_-tuned cell-free systems. As *in vivo* systems, the transcription in cell-free systems was a multienzyme reaction that synthesizes RNA from DNA templates for the later translation to proteins (Figure 2A). According to the protein expression observations discovered above, the transcriptional features of experiments on 0, 21, and 100% oxygen treatments were explored. The total RNA was extracted from cell-free systems at varied time points. The concentrations of total RNA were generally between 500 and 700 ng/μL in samples before 2 h (Supplementary Figure S10). Because there was no internal reference in cell-free systems, the sfGFP transcription level was analyzed with absolute quantification real-time PCR, in which the known sfGFP PCR product was used to make a standard curve of CT values attained from qRT-PCR and target DNA concentrations (Supplementary Figure S11). With the incubation going, the mRNA in 0 and 21% oxygen treatments followed a single-phase exponential decay rate (Figure 2B), as reported previously (Moore et al., 2018). The mRNA concentration increased first and decreased along with the incubation going, as the cell-free reagents running out. However, the transcription in 100% treatment appeared in a low state during the oxygen incubation. As the protein synthesis discussed above, both the 0 and 100% oxygen condition showed comparatively low expression. Coupled with the protein yield, the low oxygen concentration showed little influence in transcription but had a substantial effect on the translation process. However, the high oxygen concentration had a direct negative impact on transcription.

### ATP Analysis of the Cell-Free System in Different Oxygen Concentrations

Based on the results above, the direct influences on the transcription and translation regarding different oxygen concentrations are still required to be found out. The primary function of oxygen *in vivo* was the generation of ATP (adenosine triphosphate) through the aerobic respiration in aerobes (Heffner et al., 2013). In the cell-free system, ATP was an essential energy substrate for the protein synthesis that was from the initial addition or energy regeneration. ATP concentrations were then analyzed, which was considered as a critical limiting factor for cell-free systems (Moon et al., 2019) and oxygen-derived products in natural physiological systems. In the cell-free systems, 1.2 mM ATP was initially added to provide energy for the protein synthesis along with PEP (phosphoenolpyruvate) as the energy source for the ATP regeneration (Kim and Swartz, 2000). Although PEP-based cell-free systems were not dependent on the oxygen for the ATP regeneration, the usage of ATP in protein synthesis probably involved with the oxygen.

**FIGURE 3:**
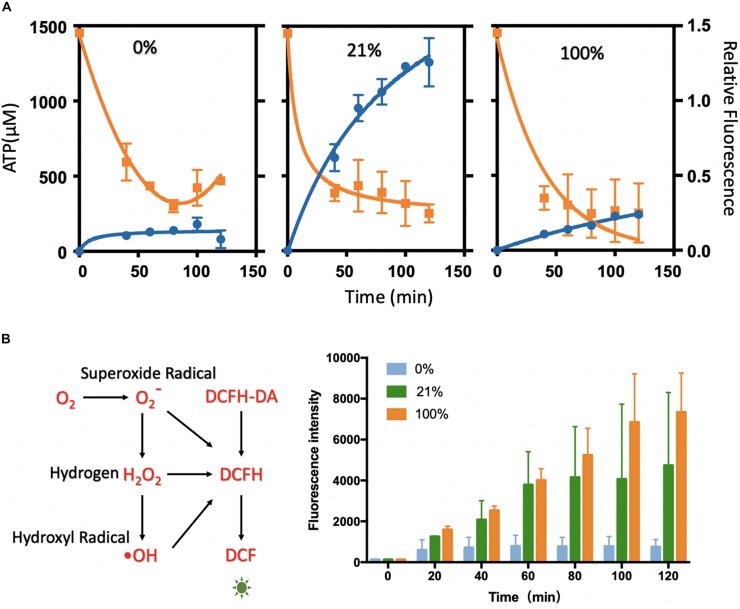
Analysis of ATP level and ROS level in the cell-free systems under different oxygen concentrations. **(A)** ATP analysis of cell-free systems between different gradients of oxygen treatments (0, 21, and 100%). The ATP concentration (orange) was decreasing rapidly along with the protein synthesis (blue). The relative fluorescence was related to Figure 1B. **(B)** The ROS level between the oxygen of 0, 21, and 100%. With the incubation, the fluorescence rose rapidly in 21 and 100%, while little changes in 0% oxygen condition. Values were calculated as the mean of biological replicates.

ATP quantification relies on a series of energy-related reactions to transfer ATP concentration to the luminescence signal. Given concentrations of ATP were used to draw the standard curve to couple the luminescence intensity with ATP concentration for the ATP quantification (Supplementary Figure S12). The ATP assay was performed at varied time points of the oxygen treatment at different levels (Figure 3A and Supplementary Figure S13). As the protein synthesis reaction going, the ATP substrates were continuously and quickly consumed after 20 min. The quick decline could be mainly attributed to the protein synthesis. In the following incubation, ATP was in a tendency of decreasing or fluctuating, and it reached a rather low level at 2 h that had limited the synthesis reactions. It seemed that ATPs were continuously regenerated from degradative PEP energy source (Kim and Kim, 2009) and exhausted gradually in 2 h. Coupled with the protein yield results, the reduction of ATP was strongly related to the synthesis process. The higher protein yield corresponded to more rapid ATP consumption. However, from the ATP analysis at varied oxygen concentrations, it seemed that oxygen was not the decisive substrate for the ATP generation or consumption.

### Reactive Oxygen Species (ROS) Analysis

According to the analysis above, ATP was the limitation substrate in the protein synthesis in all of the oxygen level. To gain more insight into the effects of the oxygen concentration on the protein synthesis, the oxygen byproducts that might affect the protein synthesis were further analyzed. The level of ROS (reactive oxygen species, O^2–^, H_2_O_2_, and OH) was investigated in the oxygen-tuned cell-free system, which was considered as the metabolic intermediate of oxygen and affecting many physiological processes (Schieber and Chandel, 2014; Ezraty et al., 2017; Reichmann et al., 2018; Zhu and Dai, 2019).

The treatments of 0, 21, and 100% oxygen were chosen for the characterization, as they were representative in oxygen-tuned CFPS. The ROS levels were characterized by DCFH-DA (2,7-dichlorodihydrofluorescein diacetate). In this assay, DCFH-DA was catalyzed to DCFH (2,7-dichlorodihydrofluorescein), which could be oxidized by ROS to form fluorescent DCF (2,7-dichlorofluorescein) (Figure 3B). In this way, the ROS levels were compared by measuring the fluorescence levels of DCF (excitation at 488 nm, emission at 525 nm). Cell-free reactions with DCFH-DA were injected into the tube-in-tube reactors for incubation without fluorescent protein synthesis. Fluorescence was measured every 20 min in 2 h incubation to reflect the amount of ROS (Figure 3B). With the increase of the oxygen concentrations in the cell-free reactions, the ROS levels increased with the incubation time. It showed a low level in 0% oxygen treatment, higher in 21% oxygen treatment, and maximum in 100% oxygen treatment along with the incubation. It seemed that the fluorescence intensity was not apparent without oxygen treating, which indicated that there was little ROS generated under this condition. Compared to 0% oxygen treatment, the fluorescence in 21 and 100% increased rapidly, indicating that ROS was generated along with the reaction, which might have some harmful effects on protein synthesis.

### Proteomics Analysis in Cell-Free Systems Between Different Oxygen Tension Treatments

**FIGURE 4:**
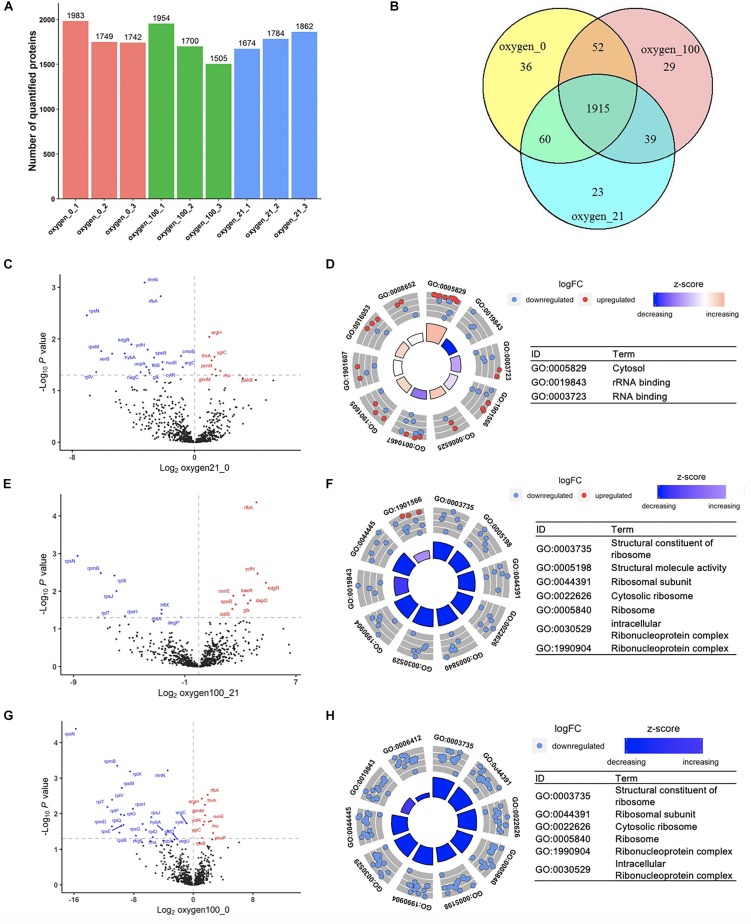
Proteomic analysis of the cell-free systems under different oxygen concentrations (Oxygen_0 represented the samples of cell-free reactions conducted in 0% oxygen, oxygen_21 in 21% oxygen, and oxygen_100 in 100% oxygen. Each treatment was in three replicates.). **(A)** The number of quantified proteins among samples. **(B)** Number of common and unique proteins among samples in Venn Diagram. **(C,D)** Volcano plot and GOCircle plot of proteins in different abundance from the comparison from 21 to 0% oxygen condition. **(E,F)** Volcano plot and GOCircle plot of proteins in different abundance from the comparison from 100 to 21% oxygen condition. **(G,H)** Volcano plot and GOCircle plot of proteins in different abundance from the comparison from 100 to 0% oxygen condition. (More details of the GOCircle annotation were shown in Supplementary Figures S23–S25).

To parameterize the protein transformation in the oxygen-tuned cell-free system, the proteomes of the CFPS system in 0, 21, and 100% after 4-h incubation were then analyzed. Each treatment was in three replicates. The analysis was completed by LC-MS/MS. Approximately 1,800–2,100 proteins were validated in each sample, and the common 1,915 proteins were identified for further analysis (Figures 4A,B). Samples in the same condition group showed high repetitive (Supplementary Figures S14–S17). A total of 52 proteins with different abundance were selected out with ANOVA (Anscombe, 1948). Most of them were related to energy metabolism, transcription, translation, and protein folding (Supplementary Figure S18).

Efficient transcription and translation were the core processes in protein synthesis. Although additional T7 operon was mainly in charge of the sfGFP synthesis in this cell-free system, the complex endogenous transcription and translation components also played an essential role (Shin and Noireaux, 2012; Garamella et al., 2016). The GO (gene ontology) analysis was used for the protein function annotation in biological process, cellular component, and molecular function. Among the identified 1,915 proteins, 812 proteins were commonly selected for further analysis. Most of them were related to energy regeneration and protein translation (Supplementary Figure S19). For biological process, it showed that 40 proteins were annotated as “translation” and 15 proteins were annotated as “protein folding,” which were enriched in cell-free systems for efficient protein synthesis. For cellular component, 62% of proteins were enriched in the term of “cytosol.” For molecular function, most proteins were considered for protein binding for ATP, RNA, NAD, ribosome, and so on (Supplementary Figure S18). From the global proteomic analysis, the cell-free system involved with sufficient transcription and translation key protein complex.

Among the oxygen treatments, 52 proteins were screened out from the samples. Among the proteins in differential abundance, most of them were related to “translation,” “rRNA binding,” and “ribosomal subunit” (Supplementary Figure S19). With the increased oxygen concentration, proteins associated with the categories mentioned above were likely to be downregulated, that there was degradation in translation-related proteins in high oxygen concentration (Figures 4C–H). Compared to 0% oxygen treatment, in the 21% treatment samples, several genes were upregulated in the term of “cytosol” (Figure 4D and Supplementary Figure S20). In particular, most of the down-regulated proteins were in terms related to “rRNA binding” and “RNA binding,” which were the key components in the translation processes. In the comparison from 100 to 21%, most proteins in the differential abundance were significantly enriched in ribonucleoprotein complex members (Figure 4F and Supplementary Figure S21), and also the proteins involved in translation. Familiar with the previous comparison, in the comparison from 100 to 0%, most proteins were also primarily enriched in ribosomal protein family as well as translation-related such as “tRNA binding” (Figure 4H and Supplementary Figure S22). According to the analysis discussed above, the higher oxygen samples showed a downregulation of translation-related proteins. Although the higher oxygen (21%) already resulted in the protein downregulation of translation-related proteins, the remaining cell-free systems were still sufficient for the protein synthesis. However, much higher oxygen (100%) would lead to further degradation that limited the protein synthesis. Therefore, the treatment by a high concentration of oxygen might lead to the degradation of translation-related proteins, which could significantly affect the CFPS processes.

## Conclusion

The unknown behind the roles of oxygen in life continues to be a challenge for the fundamental physiological science. During the past decade, efforts made in microfluidics have demonstrated it is possible to explore the oxygen physiological effects in precise oxygen concentrations for the applications of fundamental biological or medical research. However, the ineffective methods for intracellular oxygen tension detection are still the limitations in the oxygen bioprocess analysis, mainly for the gas generation and gas concentration sensing system. To address the challenges mentioned above, an emerging tool that is recently widely used for the study of gas-liquid reactions were introduced. Without the intact cells, cell-free reactions are more likely to chemical reactions, where the substrate concentrations involved in bioprocess are concentrations in liquids, avoiding the limitation from the physical measurement.

Cell-free systems were employed for the analysis of the oxygen-tuned protein synthesis, coupled with a tube-in-tube reactor. The tube-in-tube reactor is regarded as a powerful tool for studying the gas-liquid reaction system, which allows gas to penetrate rapidly into the liquid in about 30 s. It could precisely control and alter the oxygen tension gradients. From this measurement platform, there appeared a maximum yield in the 21% oxygen condition, as the natural atmosphere condition. Meanwhile, no <4% oxygen tension can provide sufficient oxygen for the protein synthesis. Further analysis in mRNA, ATP, ROS, and proteome was conducted for the study of the internal connection between oxygen concentration and protein synthesis. The target mRNA in cell-free reactions seemed that there was little effect in low oxygen concentration (0%) but repressive in high concentration (100%) for nearly no transcription. With the protein synthesis, ATP showed a sharp degradation in the early protein synthesis process. Along with the increased oxygen concentration, ROS levels in cell-free reactions showed a related increase. Many translation-related proteins were downregulated in higher oxygen condition, which was of significant influence in protein synthesis. That is, a high concentration of oxygen seemed to result in a degradation of translational-related proteins and therefore limit the protein synthesis.

Oxygen-tuned protein synthesis machinery could synthesize proteins at a certain speed on demand by the regulation of the oxygen level. Coupled with great gas transfer performance in the tube-in-tube reactor and flexible CFPS system, this platform would be further well-adopted to explore gas-regulated biological cellular processes.

## Data Availability Statement

The datasets generated from this study are available in request to the corresponding authors: YL (yuanlu@tsinghua.edu.cn) and JZ (jiszhang@tsinghua.edu.cn). The mass spectrometry proteomics data have been deposited to the ProteomeXchange Consortium (http://proteomecentral.proteomexchange.org) via the iProX partner repository (Ma et al., 2019) with the dataset identifier PXD018058.

## Author Contributions

YL and JZ conceived the project, supervised the research, assisted in analyzing data, preparing the figures, and writing the manuscript. XL and CZ designed and performed the experiments, analyzed the results, prepared the figures, and wrote the manuscript. SZ and HD assisted in bioinformatic analysis.

## Conflict of Interest

The authors declare that the research was conducted in the absence of any commercial or financial relationships that could be construed as a potential conflict of interest.
