# Supplementary material for: O2-Tuned Protein Synthesis Machinery in Escherichia coli-Based Cell-Free System
Source: Front Bioeng Biotechnol. 2020 Apr 9;8:312. doi: 10.3389/fbioe.2020.00312 (PMC7160232; doi:10.3389/fbioe.2020.00312)
Supplement: Supplementary file 1 [file Table_1.docx]

**Supplementary Material**

**O_2_-tuned protein synthesis machinery in *Escherichia coli*-based cell-free system**

Xiaomei Lin^1#^, Caijin Zhou^2#^, Songbiao Zhu^3^, Haiteng Deng^3^, Jisong Zhang^2*^ & Yuan Lu^1*^

^1^Key Laboratory of Industrial Biocatalysis, Ministry of Education, Department of Chemical Engineering, Tsinghua University, Beijing, China.

^2^The State Key Lab of Chemical Engineering, Department of Chemical Engineering, Tsinghua University, Beijing, China.

^3^MOE Key Laboratory of Bioinformatics, Center for Synthetic and Systematic Biology, School of Life Sciences, Tsinghua University, Beijing 100084, China.

^#^These authors contributed equally to this work.

^*^Corresponding author. Email: yuanlu@tsinghua.edu.cn (Y.L.); jiszhang@tsinghua.edu.cn (J.Z.)

**Supplementary** **Methods**

**Fluorescent protein expression device based on tube-in-tube reactor**

The fluorescent protein expression device is mainly composed of Harvard pump (Harvard PHD, Holliston, USA), tube-in-tube reactor, mass flow controllers (Sevenstar, Beijing, China) and a heating bath (Lab companion, Seoul, Korea). The tube-in-tube reactor was established by a highly permeable Teflon AF-2400 inner tube with an inner diameter of 0.6 mm enclosed by a polytetrafluoroethylene (PTFE) outer tube (outer diameter: 3.17 mm). The ﻿Teflon AF-2400 inner tube allowed the liquids reagents steadily staying or flowing, but highly permeable for gas. The cell-free reactions were assembled on ice and then injected into the inner tube for protein synthesis. By regulating the flow rates of oxygen and nitrogen separately, the device was able to control the oxygen microenvironment of cell-free reactions. The tube with cell-free reactions was incubated in a heating bath at 37℃ (Lab companion, Seoul, Korea).

**Sampling process of protein at different residence time**

The green fluorescent protein was used as a reference system to study CFPS reaction process in the tube-in-tube reactor with different experimental conditions, which is shown in Supplementary Figure 1. The gas flowed into the outer impermeable polytetrafluoroethylene (PTFE) tube with a diameter of 3.175 mm. The flow rate of gas was controlled by a precise mass flow controller (Sevenstar, Beijing, China). Due to the good permeability of the inner tube, the oxygen in the outer tube quickly dissolved in the reaction substances and participated in protein synthesis. The reaction substrates were injected into the inner tube by a microsyringe, then 20 microliters of air was injected to propel the reactant completely into the inner tube. A Harvard syringe pump (Harvard PHD, Holliston, USA) loaded with water was used to push the reaction substrates flowing through the inner tube at a certain flow rate. Using the injection/extraction function of Harvard syringe pump, 20 microliters of reactants were pushed out from the inner tube collected to EP tube every 20 minutes in 2 hours. The collected samples were immediately placed in a refrigerator at - 80 ℃ to stop the protein synthesis for further analysis. The chart of sampling is shown in Supplementary Figure 2.

**Proteomic analysis**

*Sample preparation*

The same volume of cell-free reactions that incubated with 0%, 21%, and 100% oxygen for 4 hours were separated by SDS-PAGE (12% separating gel). Samples were diluted four times with phosphate-buffered saline (PBS). The dilutions were heated at 98 ℃ with 6 x protein loading buffer (TransGen Biotech, DL101-02) and then loaded onto the polyacrylamide gel (12% separating gel), running at running buffer (Tris-glycine-SDS) at 140 V, 200 mA for 1 h. The loaded gels were sent to the Center of Biomedical Analysis, Tsinghua University, for LC-MS/MS analysis.

*Statistical rationale*

The acquired raw data were processed using MaxQuant, and the database was used (Uniport, *Escherichia coli* K12). R programs were used to screen out the differential proteins (P<0.05). Enrichment analysis including Gene Ontology (GO) annotation was performed using DAVID 6.8 (The Database for Annotation, Visualization, and Integrated Discovery) tools with the total *Escherichia coli* genome information as the background. The GO annotations were visualized with R packages.

**Supplementary Figures**

**
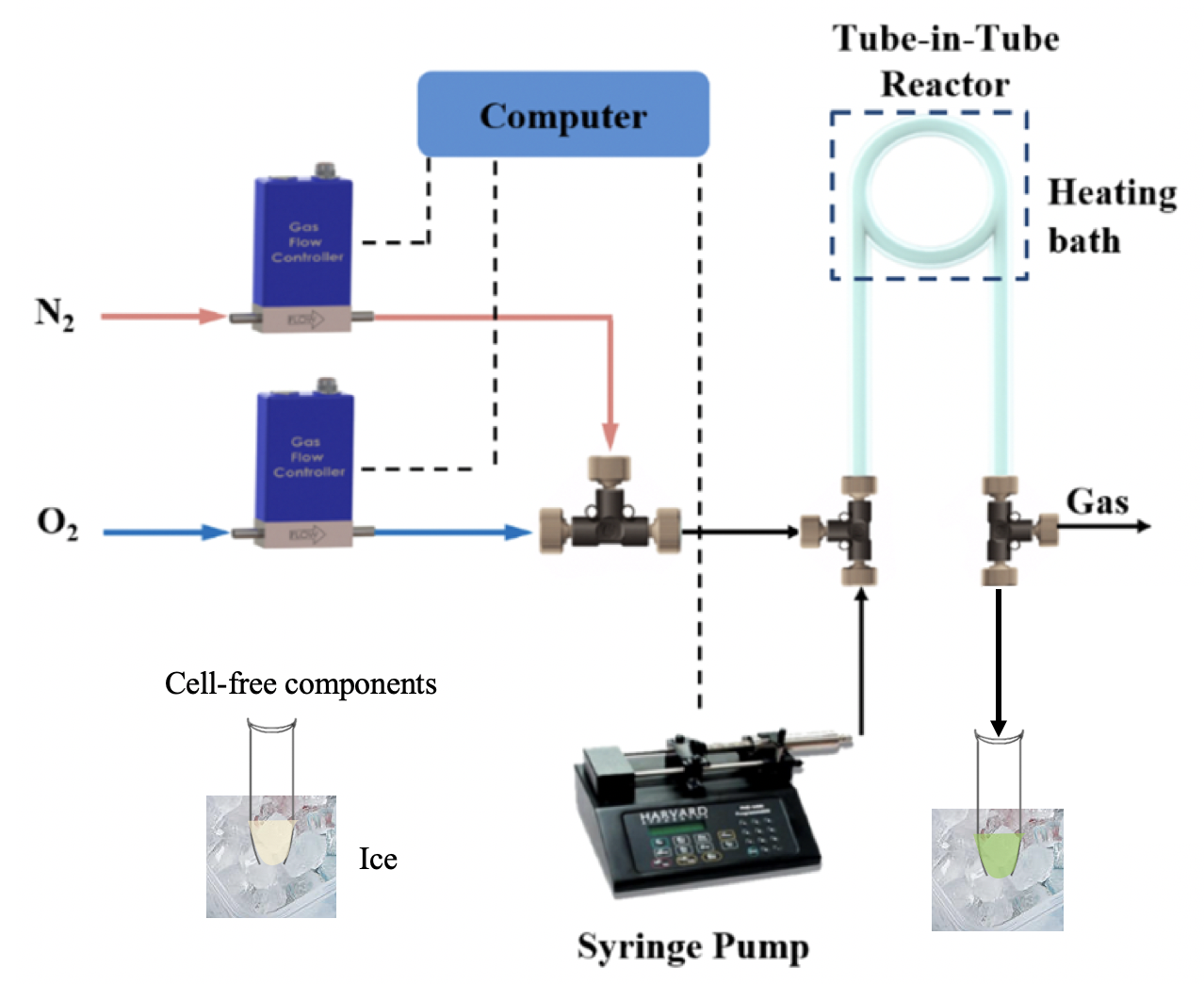
**

**Supplementary Figure 1. Schematic of cell-free protein expression system based on the tube-in-tube reactor.** The cell-free systems were assembled on ice and injected into the inner tube for incubation. The reactants were pushed out every 20 min in the first 2 hours.


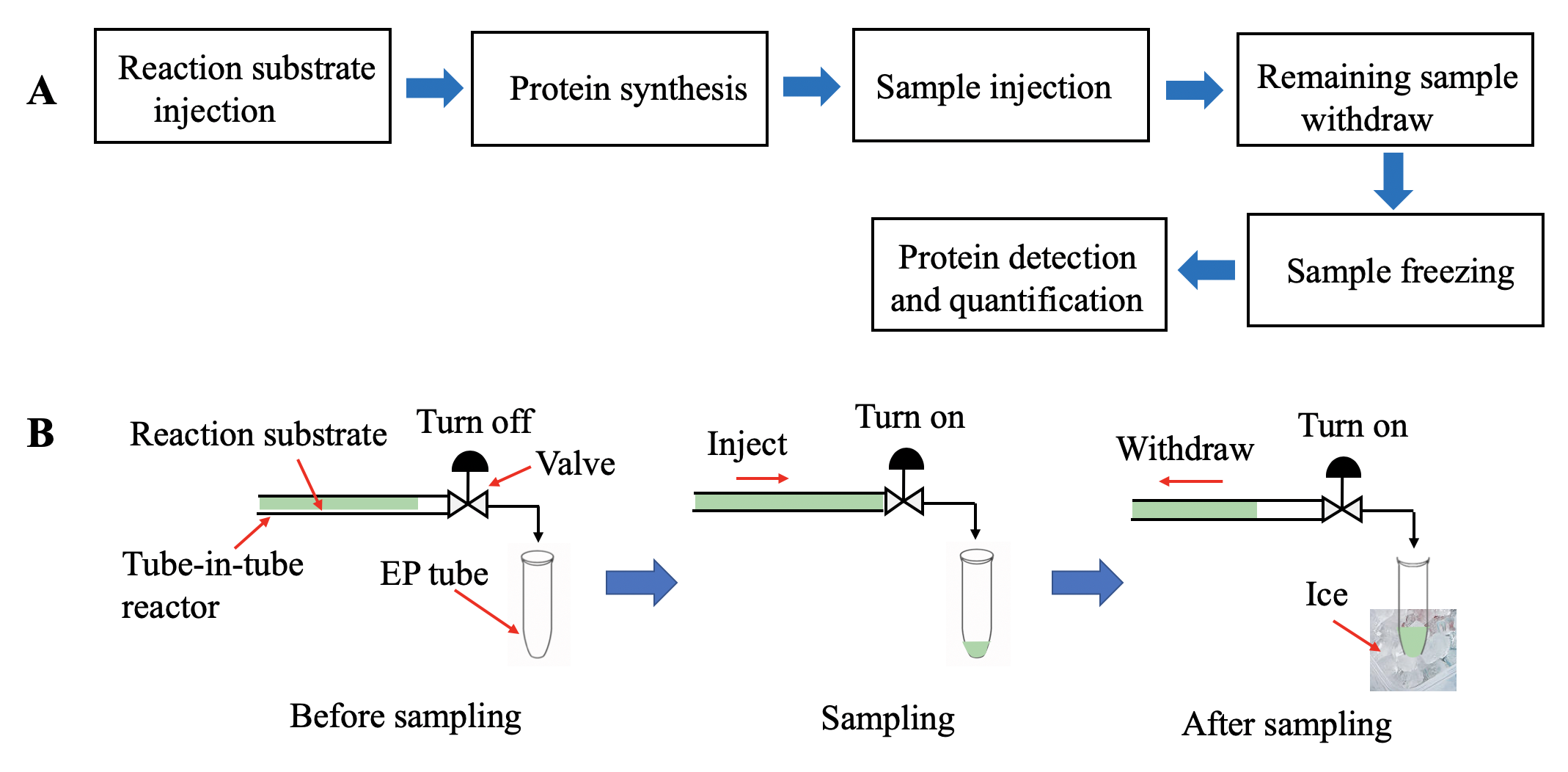


**Supplementary Figure 2.** **The operational process of the cell-free protein synthesis platform in the tube-in-tube reactor.** (A) The chart of the protein sampling process at different residences. (B) The detailed diagram of the protein sampling process.


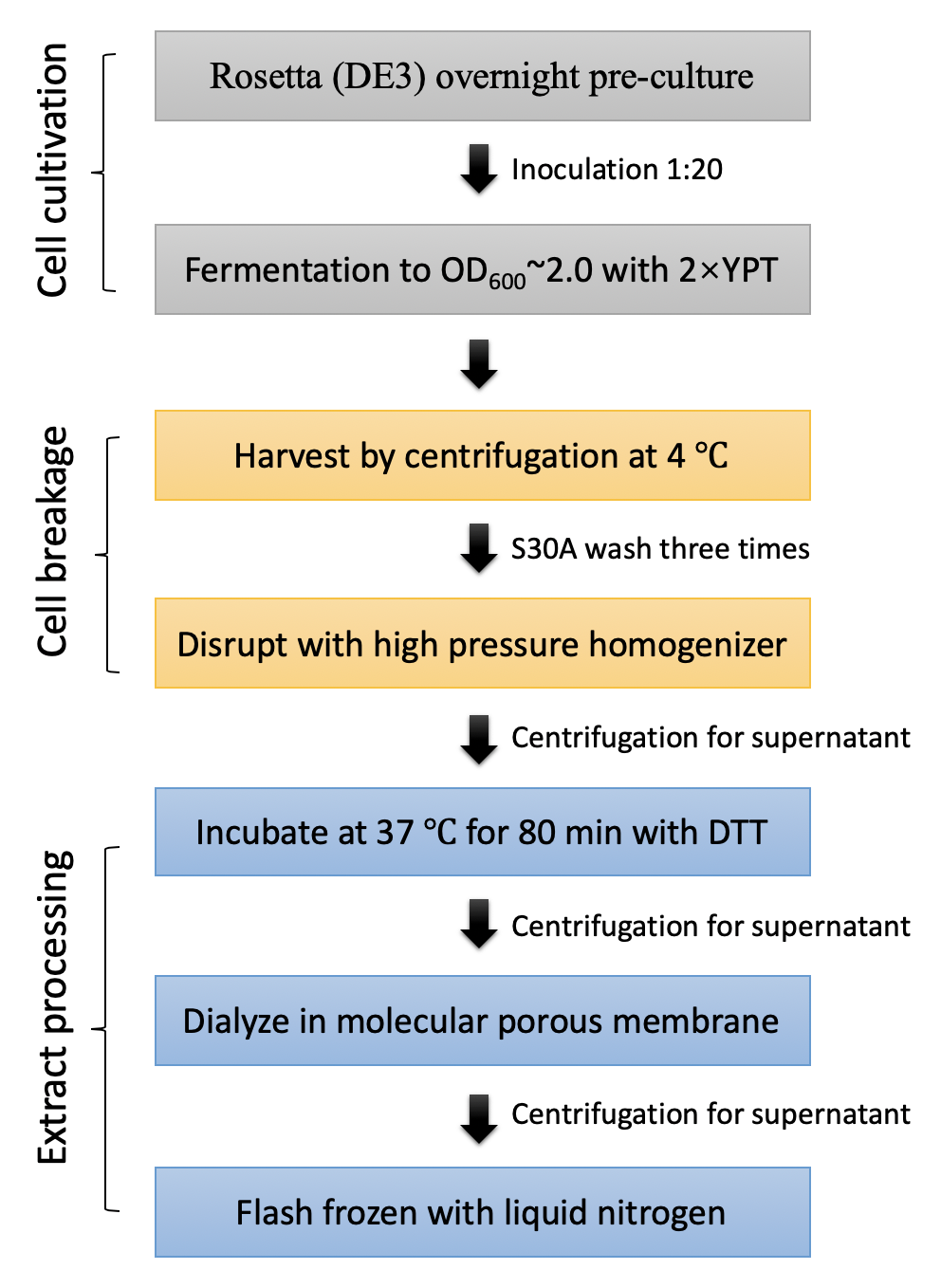


**Supplementary Figure 3. The workflow of the cell-extract preparation.** *E. coli* Rosetta (DE3) cells were incubated overnight and inoculated into fresh 2 x YPT culture for fermentation. After incubation, cells were then harvested by centrifugation. The cells were resuspended in S30A for disruption after washed with S30A buffer for three times. The supernatant of lysis was incubated at 37 ℃ for 80 mins with DTT. After being centrifuged, the supernatant was dialyzed in molecular porous membrane tubes, flash-frozen, and stored at -80 ℃.


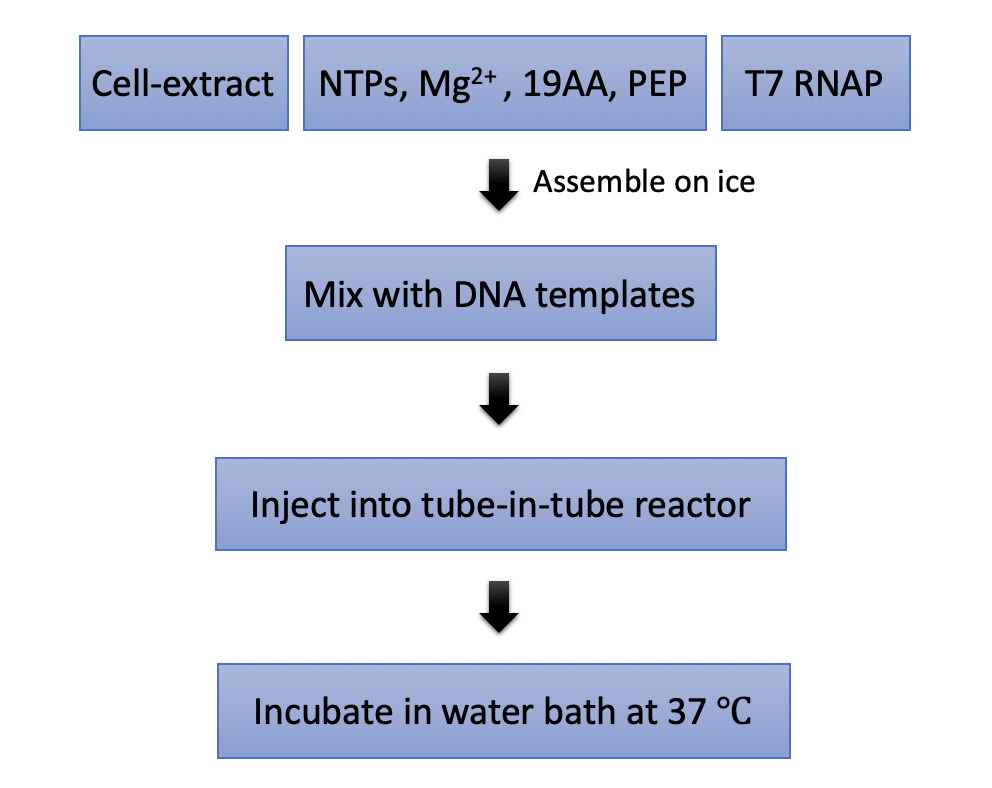


**Supplementary Figure 4. Workflow for the preparation of cell-free reactions.** Cell-free reaction components (cell extract, NTPs, Mg^2+^, 19 amino acids, PEP, and T7 RNA polymerase) were assembled on the ice. After mixing with DNA templates that express sfGFP, the cell-free protein expression systems were injected into the tube-in-tube reactor for the protein synthesis at 37 ℃ with water bath.


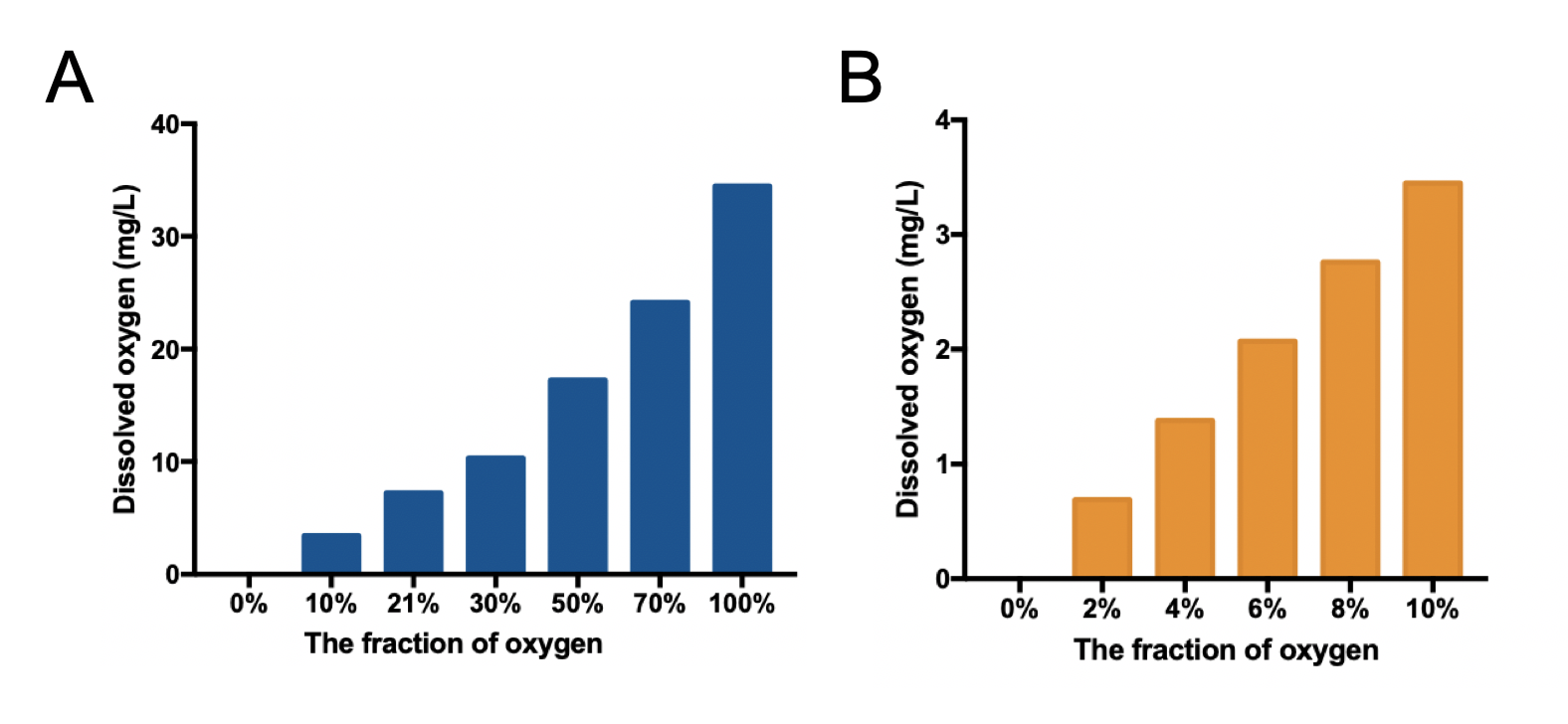


**Supplementary Figure 5. The dissolved oxygen in the tube.** (A) The dissolved oxygen concentrations with oxygen tensions of 0, 10%, 21%, 30%, 50%, 70%, and 100% at 37 ℃. (B) The dissolved oxygen concentrations with oxygen tensions of 0%, 2%, 4%, 6%, 8%, and 10% at 37 ℃.


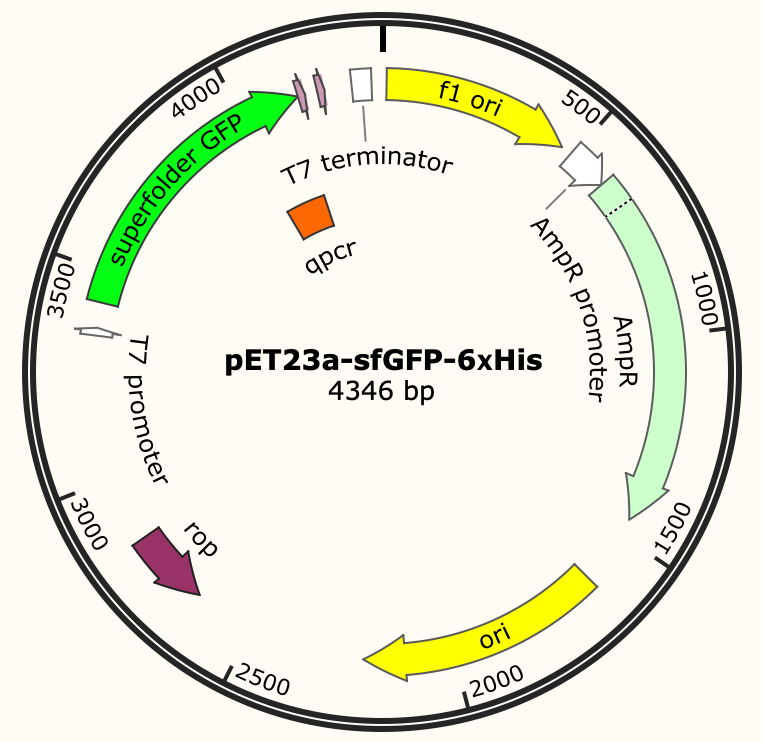


**Supplementary Figure 6. The plasmid map of sfGFP (super fold green fluorescent protein).** The sfGFP was regulated under the bacteriophage derived T7 promoter and terminator in the pET-23a backbone. The last 150 bp of sfGFP were designed for the qPCR (Real-time Quantitative PCR).


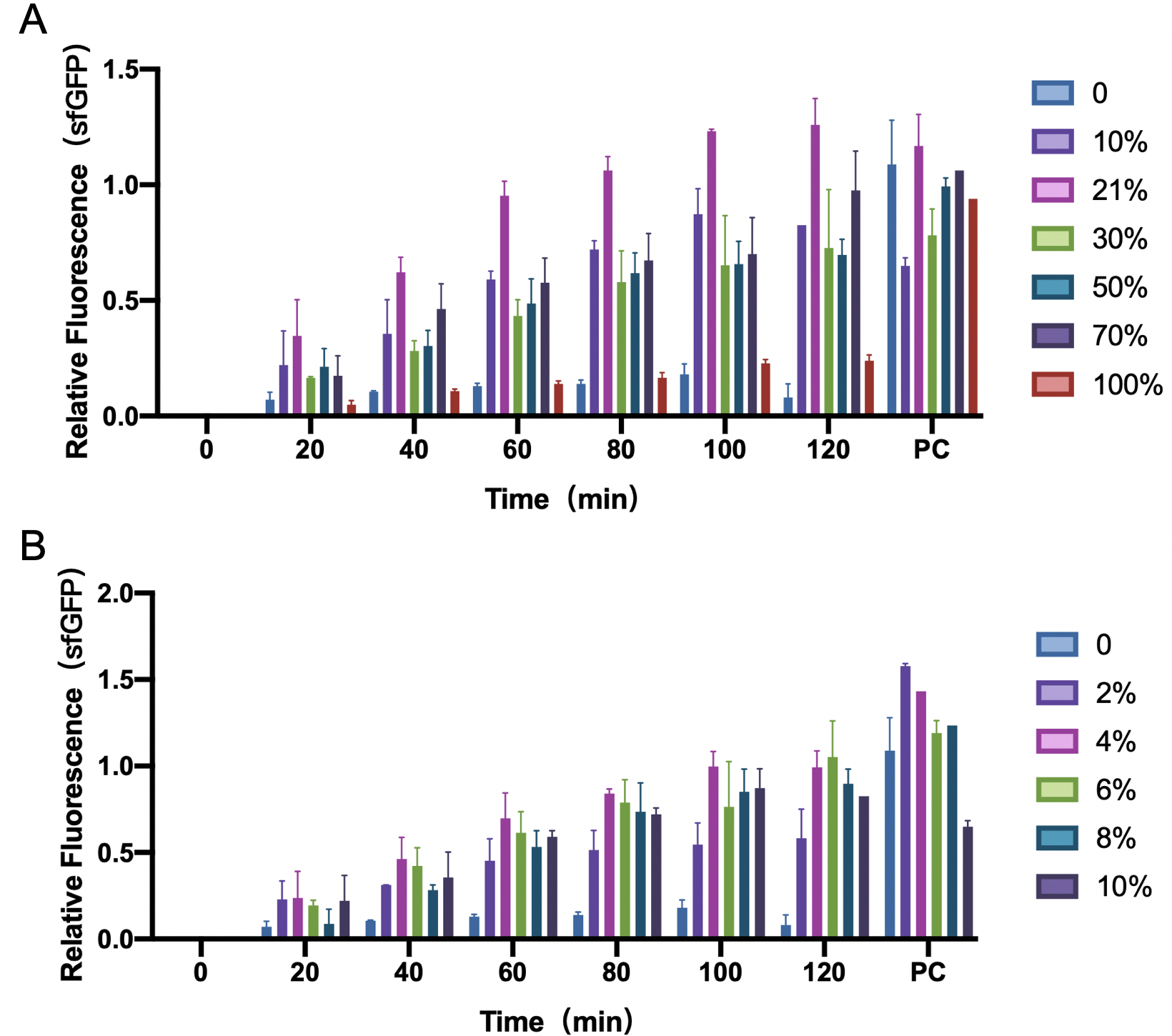


**Supplementary Figure 7. Normalized fluorescence values of synthesized sfGFP proteins.** (A) Normalized fluorescence of sfGFP incubated from cell-free systems in the tube-in-tube reactor with oxygen tensions of 0, 10%, 21%, 30%, 50%, 70%, and 100% in 2h and 37℃, that was relative to Figure 1B. (B) Normalized fluorescence of sfGFP incubated from cell-free systems in the tube-in-tube reactor with oxygen tensions of 0%, 2%, 4%, 6%, 8%, and 10% in 2 h and 37℃, that was relative to Figure 1C. Moreover, the protein synthesis of PC (positive control) was performed in the regular tube (Eppendorf tube) under the air condition (21%, 8 mg/ml).


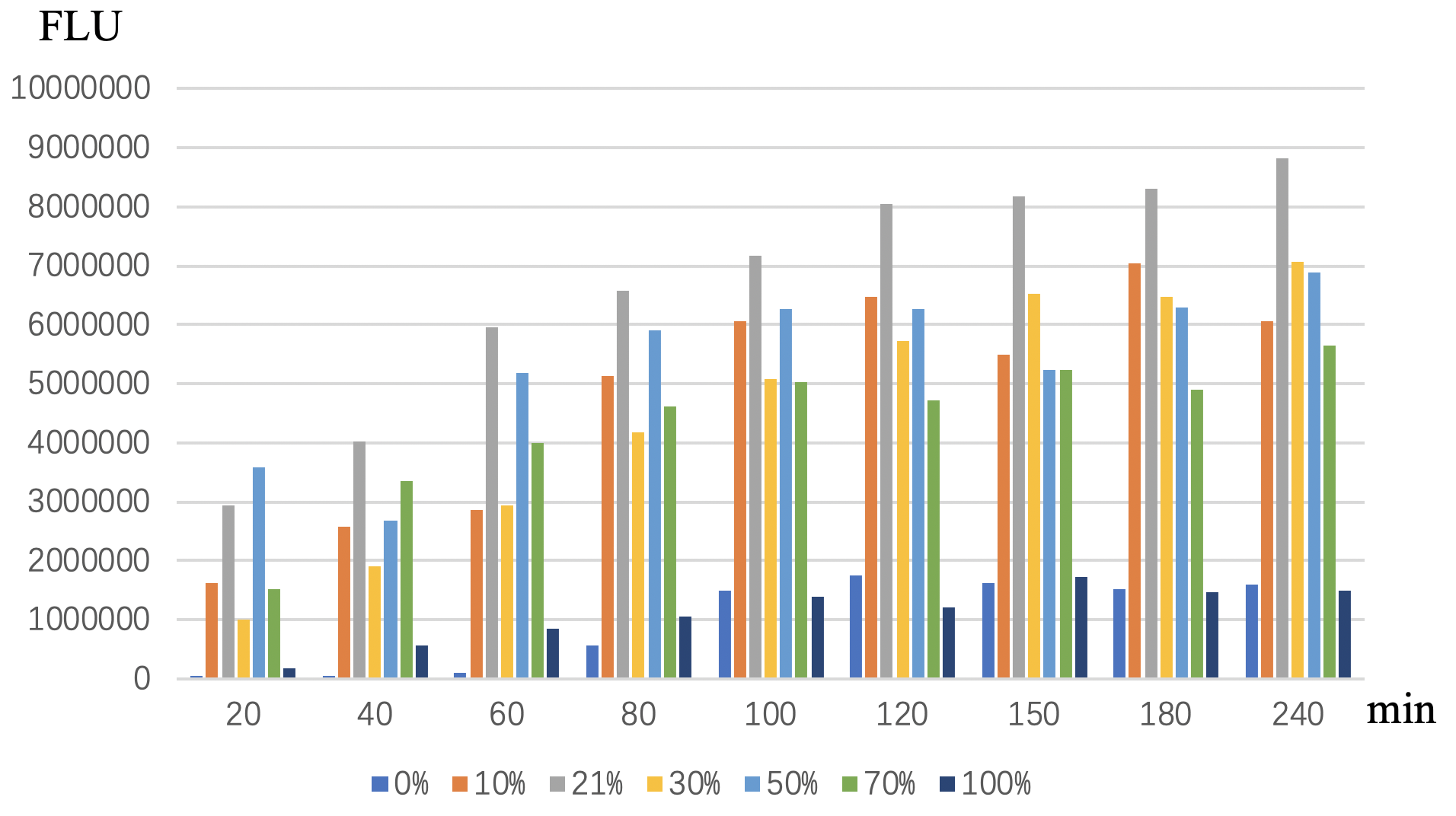


**Supplementary Figure 8. The cell-free synthesis reactions were initially conducted in 4 h (240 min).** The intensity of fluorescence was increased rapidly and reached a maximum within 2 h.


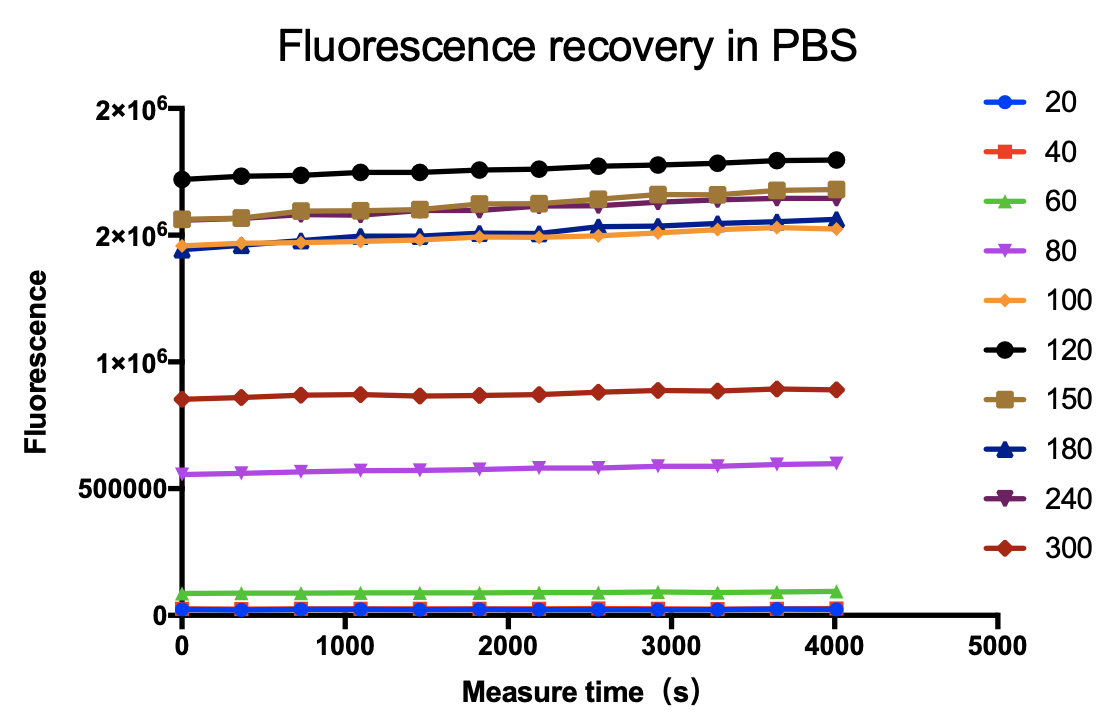


**Supplementary Figure 9. Fluorescence recovery of 0% oxygen treatment in PBS.** Fluorescence recovery of sfGFP in PBS of 0% oxygen treatments (20, 40, 60, 80, 100, 120 min) for 1h in plate reader with shaking. Oxygen was an essential and sensitive substrate of sfGFP fluorescence. It showed that fluorescence was recovered in the first measurement because there was little change in the fluorescence intensity of samples.


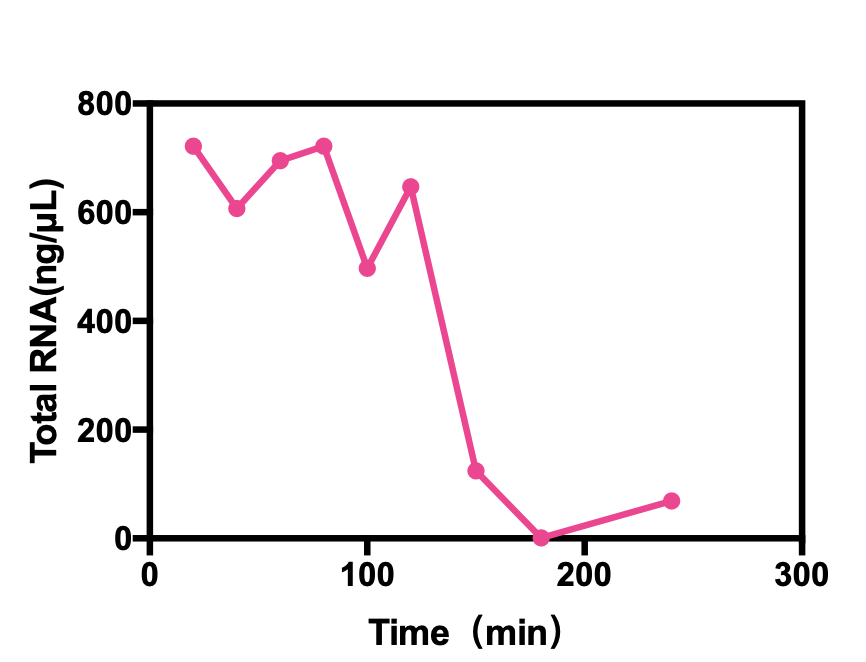


**Supplementary Figure 10. Total RNA concentrations.** The concentrations of total RNA that were extracted from cell-free reactions at varying time points. The RNA levels were approximately 600-800 ng/μL within the 2-h incubation. However, there was a decline after 2-h incubation.


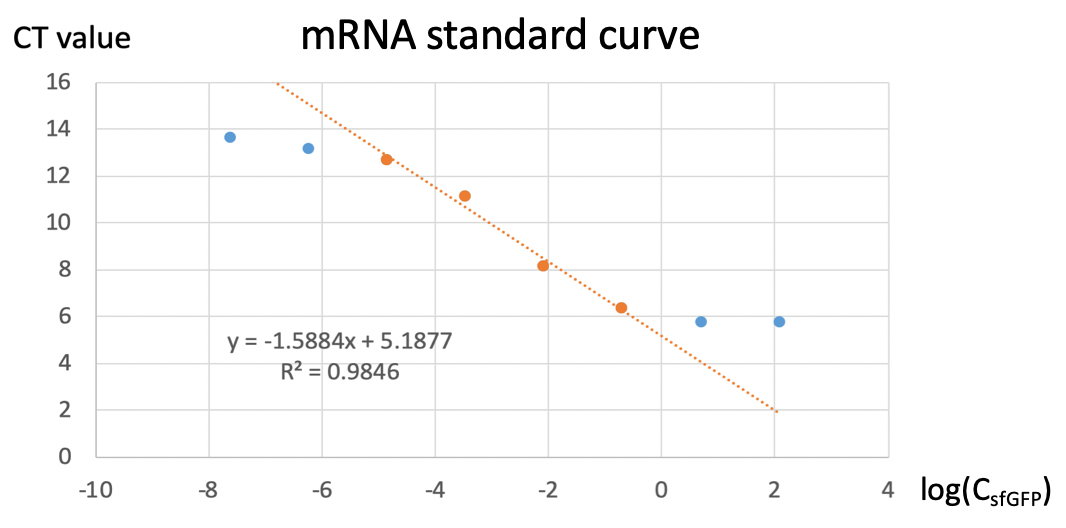


**Supplementary Figure 11. The concentration of sfGFP DNA concentration and CT value standard curve.** PCR product in known concentrations was employed for drawing the standard curve. The CT value read by instrument (ABI 7300 Real-Time PCR System) has a linear correlation with the log of sfGFP DNA concentration. It seemed that only the initial concentration of sfGFP DNA between 0.0078 ng/μl and 0.5000 ng/μl was in the linearity range. The linear equation was y = -1.5884x + 5.1877 (y for CT value, x for the log of sfGFP DNA concentration).


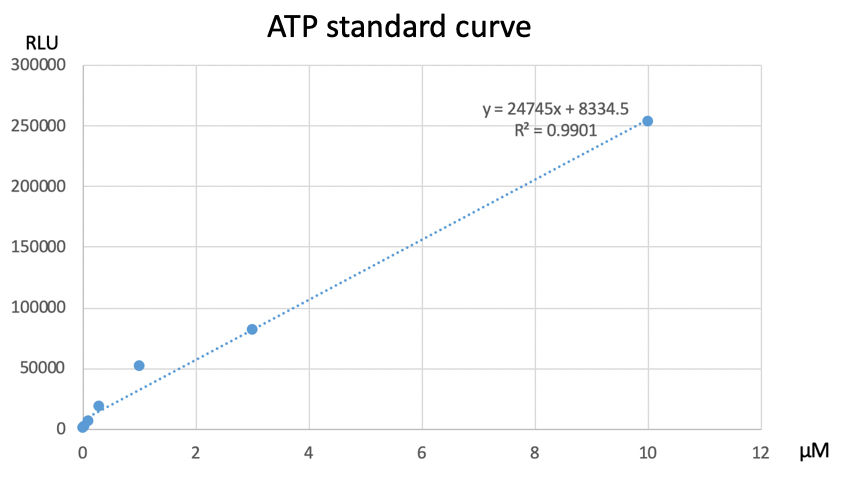


**Supplementary Figure 12. The standard curve between ATP concentration and luminescence.** A series of ATP solutions in known concentrations were employed for coupling the luminescence and ATP concentrations of the ATP assay kit (Beyotime, S0026). The linear equation was y=24745x+8334.5 (y for luminescence and x for ATP concentration).


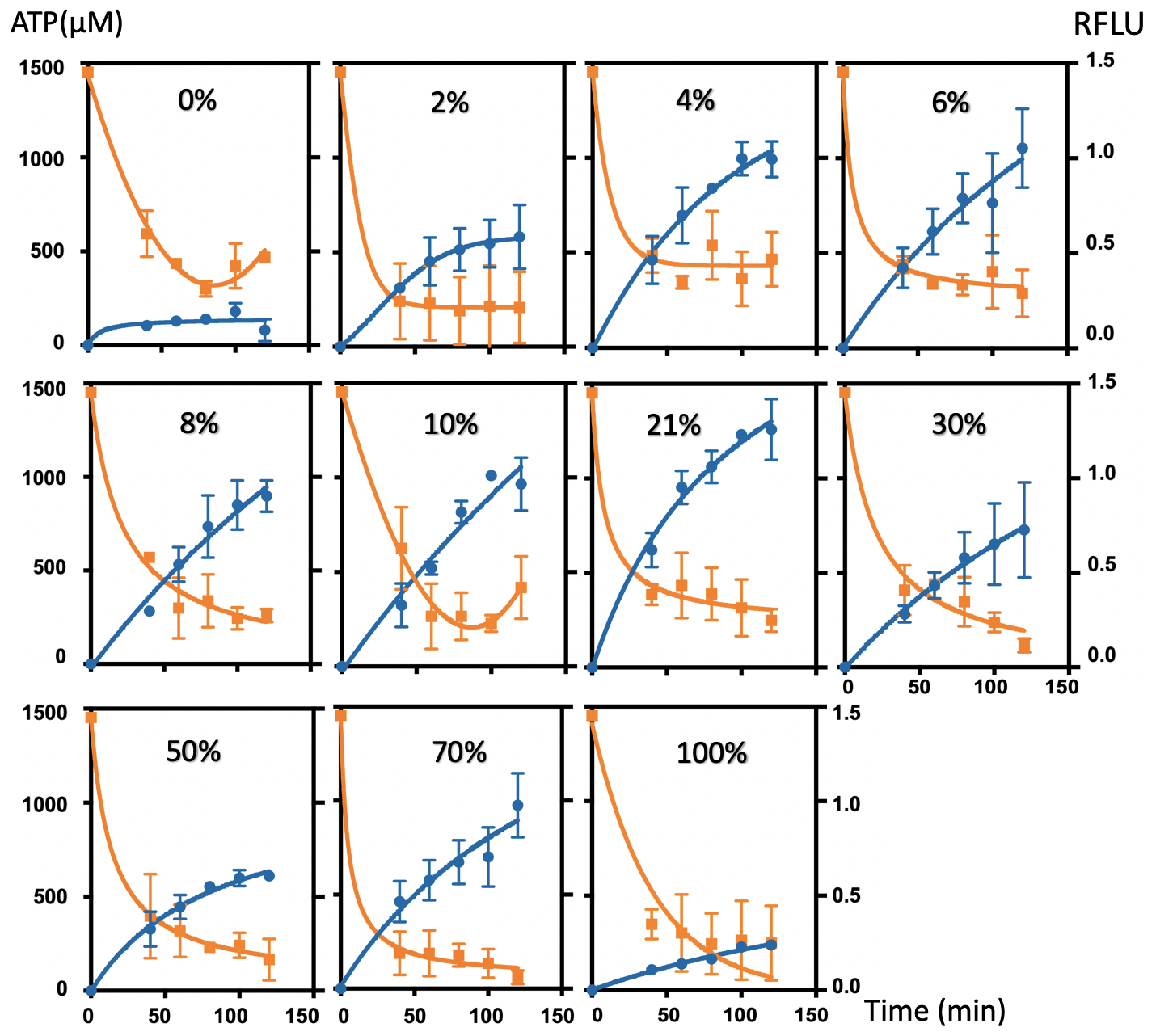


**Supplementary Figure 13. ATP analysis of cell-free systems between different gradients of oxygen treatments.** The ATP concentration (orange) was decreasing rapidly along with the protein synthesis (blue). The relative fluorescence was related to Figure 1B.


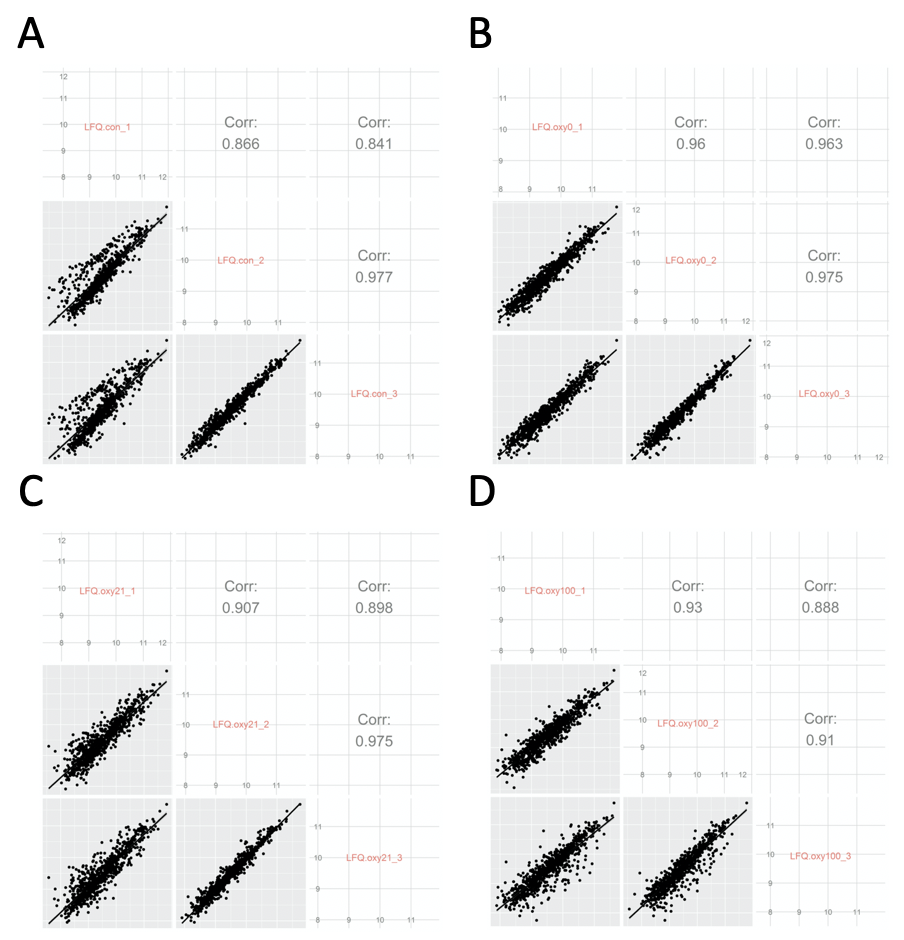


**Supplementary Figure 14. The correlation between three replicates of three treatments in protein intensity. (A)** The protein intensity correlation between three replicates of a negative control (initial cell-free reaction without protein synthesis) (**B)** The protein intensity correlation between three replicates of samples incubated in the 0 % oxygen. **(C)** The protein intensity correlation between three replicates of samples incubated in the 21 % oxygen. **(D)** The protein intensity correlation between three replicates of samples incubated in the 100 % oxygen. Because of the instrumental error, there was a sample that was not so matched to the other two samples in each treatment. For the result gained from the raw data, they needed to be normalized.


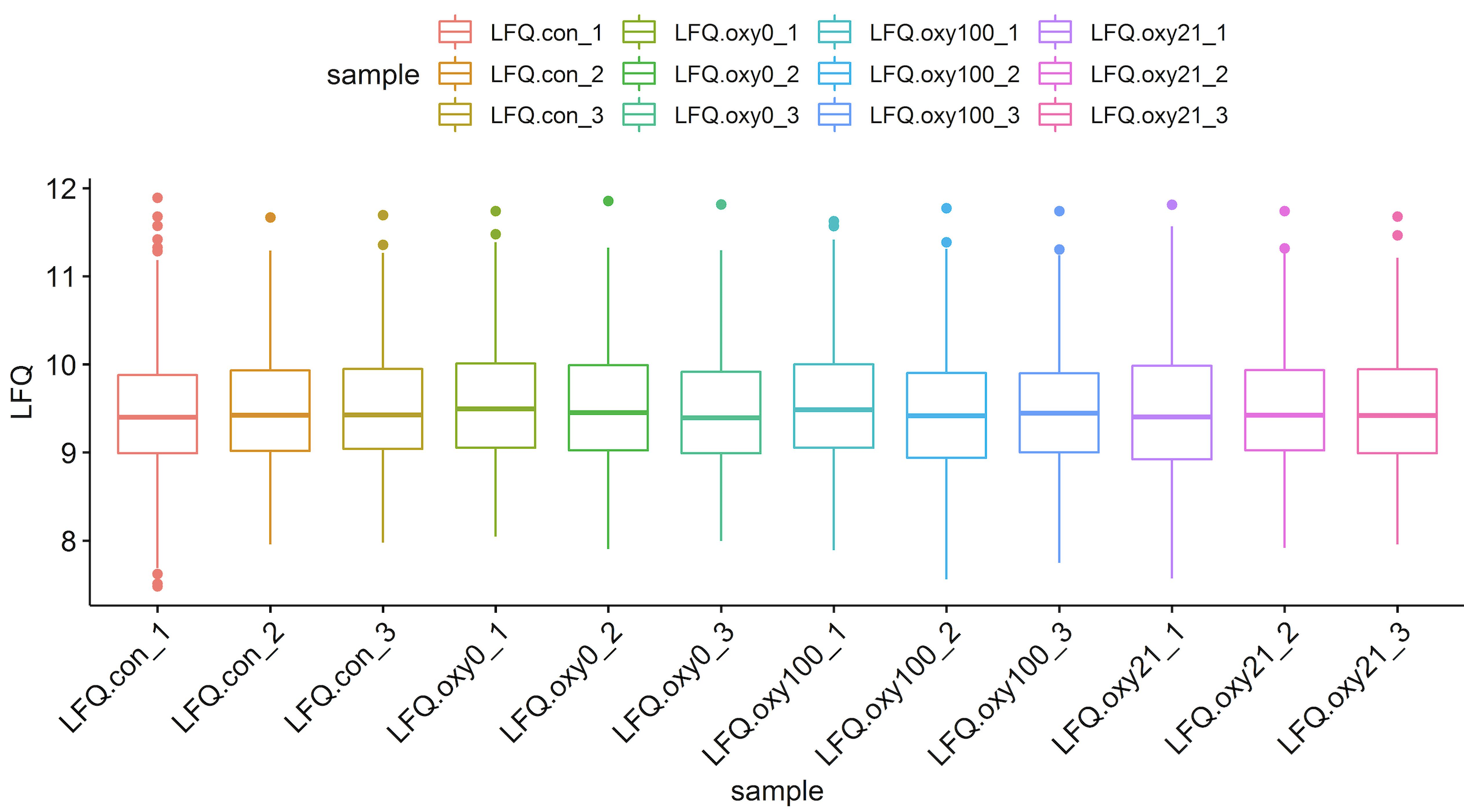


**Supplementary Figure 15. The box-plot of samples.** The box-plot showed that the protein intensity of samples was in a similar distribution.


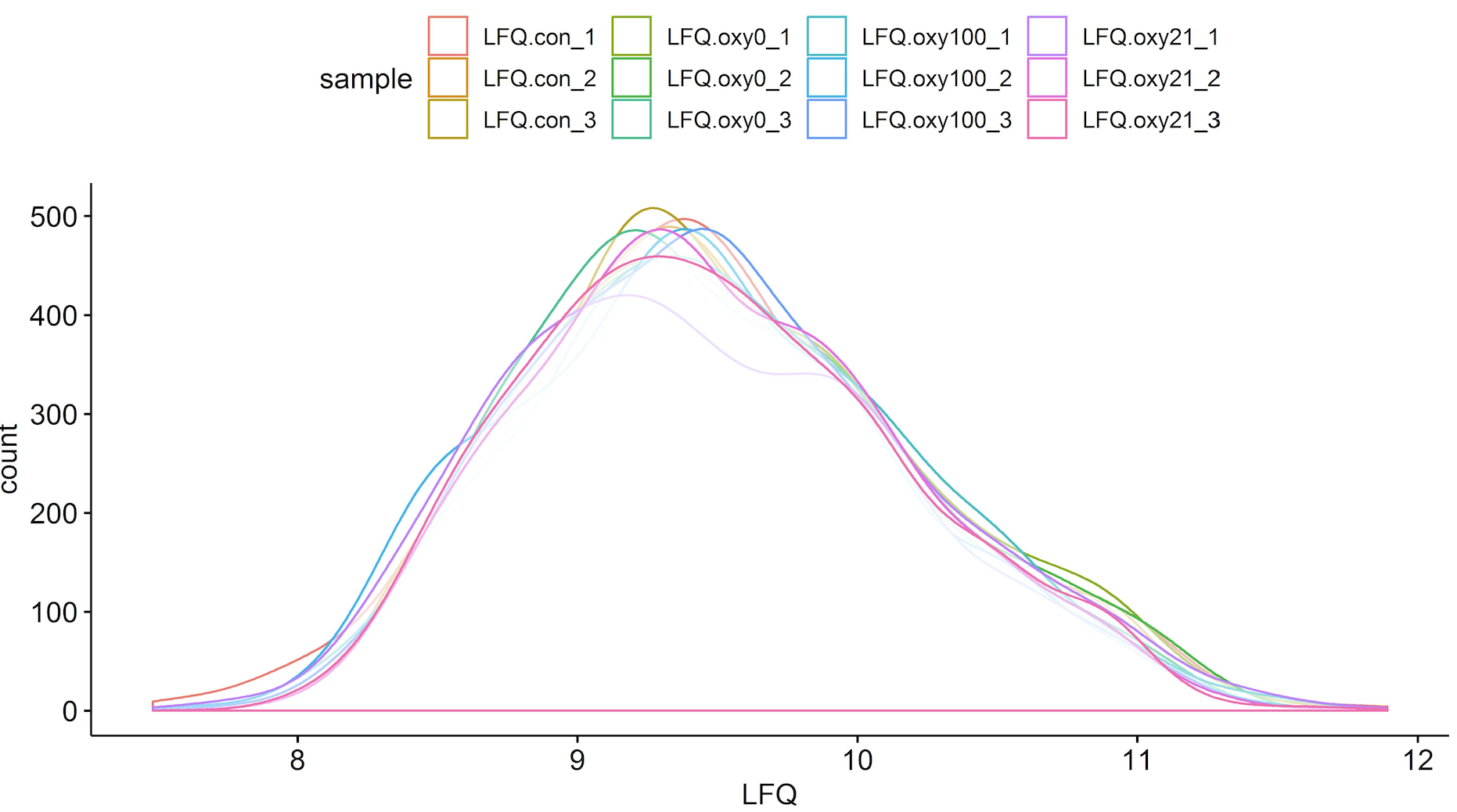


**Supplementary Figure 16. The normal distributions of samples.** The normal distribution showed that the samples were in a disposition.


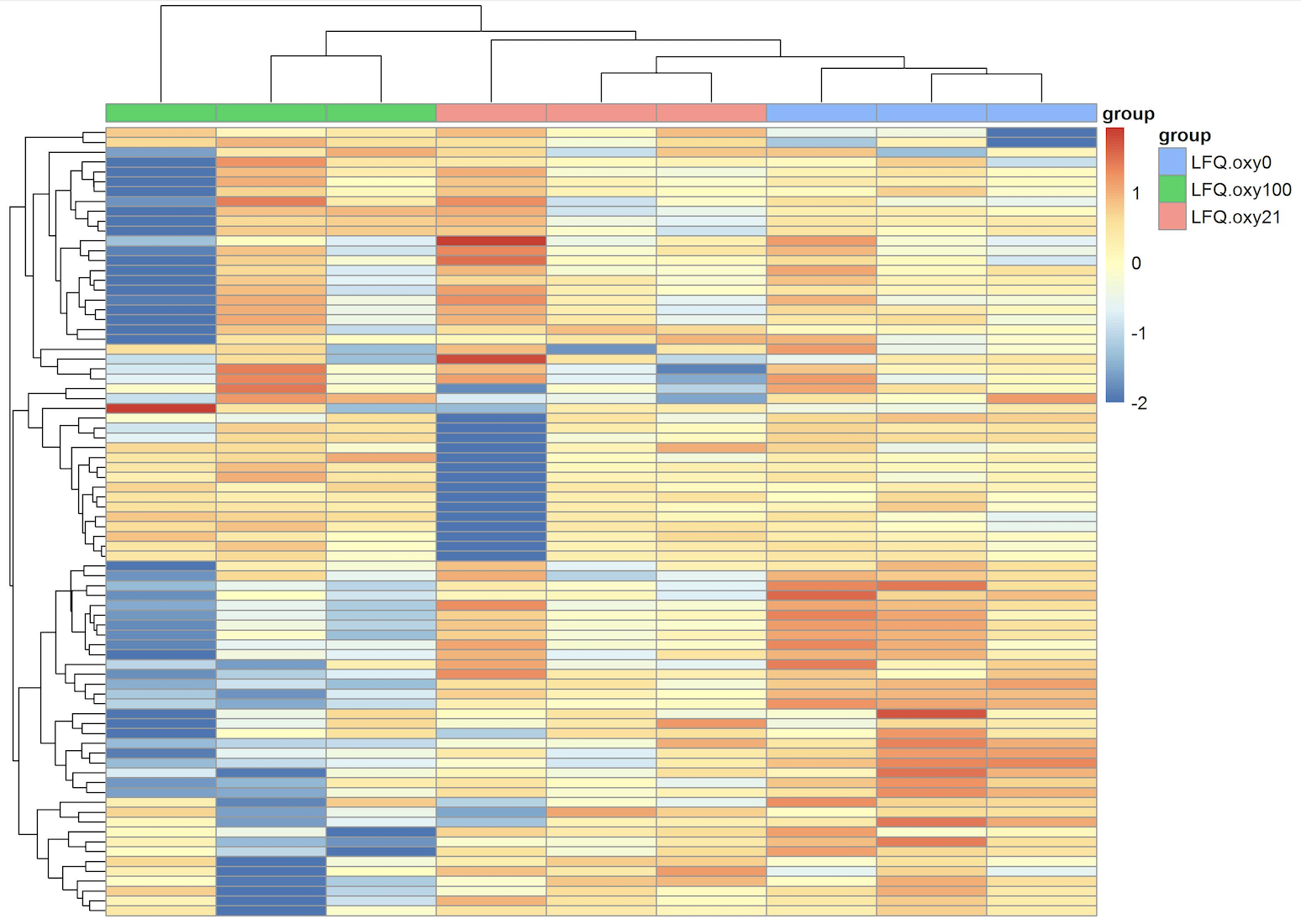


**Supplementary Figure 17. Cluster analysis of samples.** Samples of three replicates among 0%, 21%, and 100% oxygen treatments showed the homology.


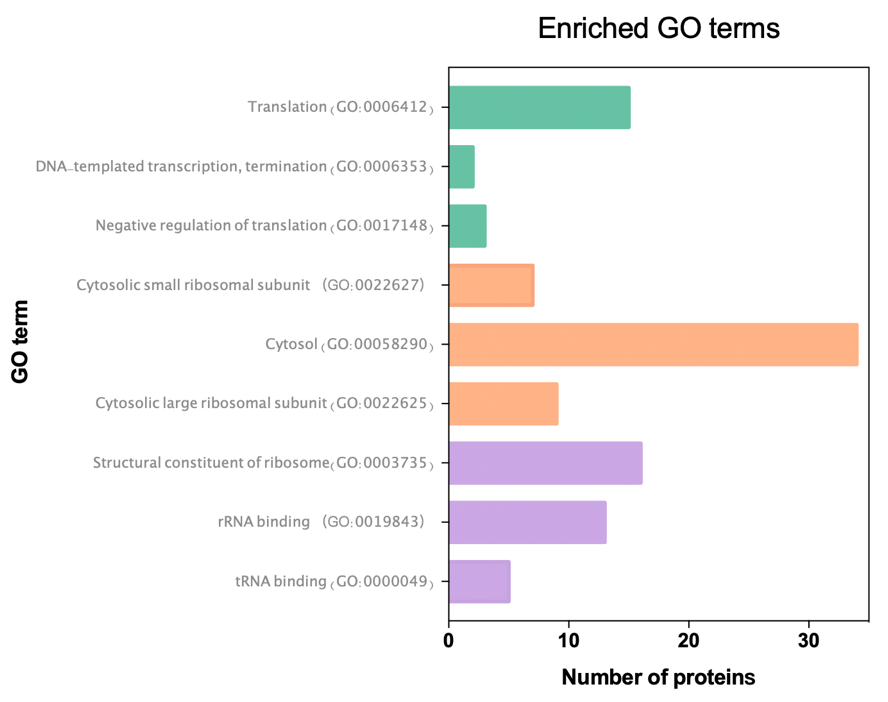


**Supplementary Figure 18. GO annotation of 52 proteins of differential abundance.** For the molecular function annotation (green), most of proteins were related to translation. For the cellular component annotation (orange), ~32% of proteins were annotated as ribonucleoprotein complex members. For the biological process annotation (purple), the proteins were significantly enriched in the terms of translation processes.

**Supplementary Figure 19. GO annotation of the common 812 proteins in all samples.** Many of the proteins were enriched in terms of protein/ATP/Mg^2+^/NAD/RNA binding, ATP binding in biological process (purple). Most of proteins were enriched in terms of cytosol, cytoplasm, and membrane in molecular function. In cellular function, proteins were enriched in terms of translation.


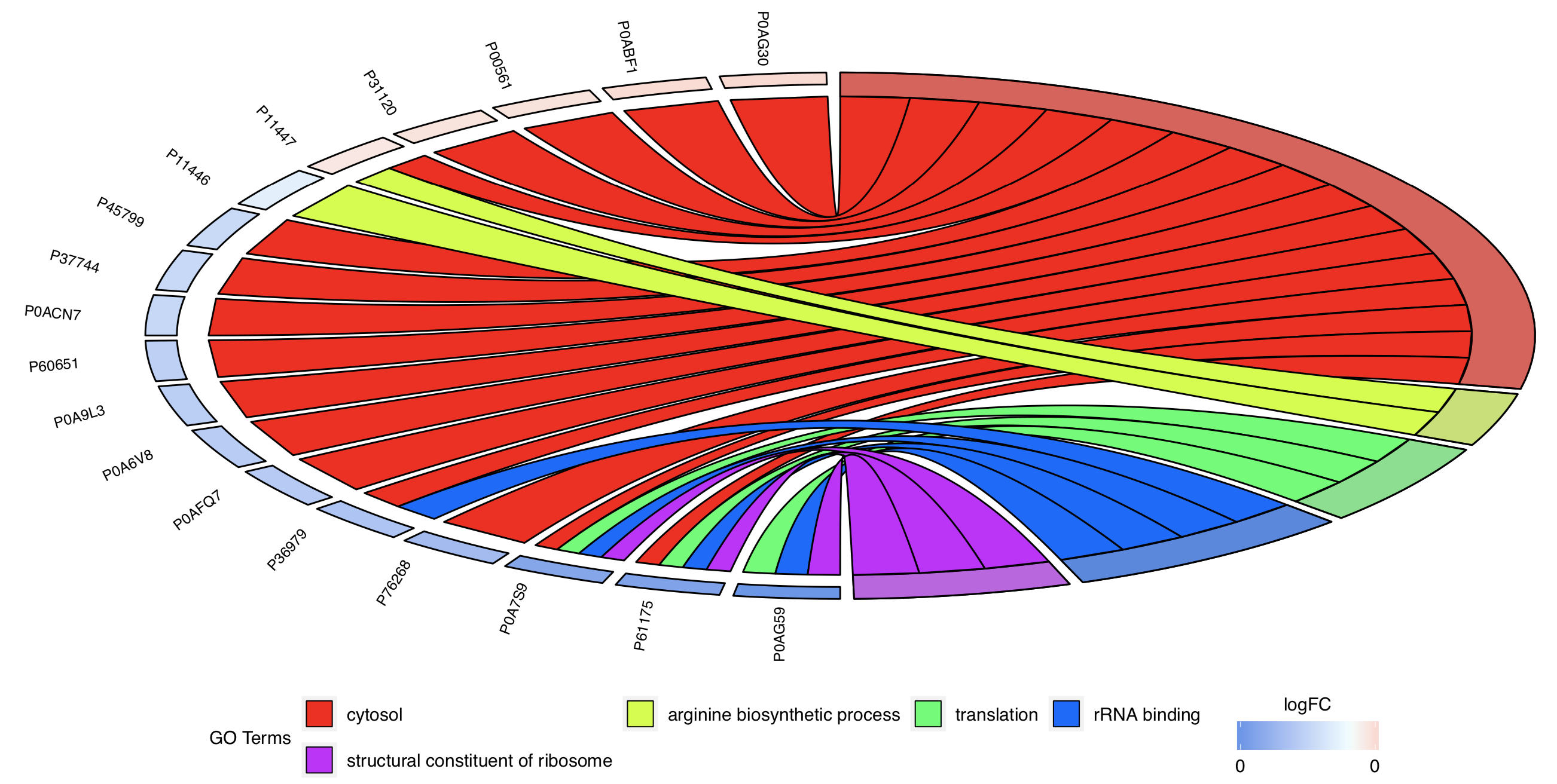


**Supplementary Figure 20. Enriched terms of differential proteins between the samples of 21% and 0% oxygen incubation.** Most of differential genes were in the term of “cytosol”. Moreover, several differential expression genes were correlated to the translation process. The protein information was shown in Table S1.


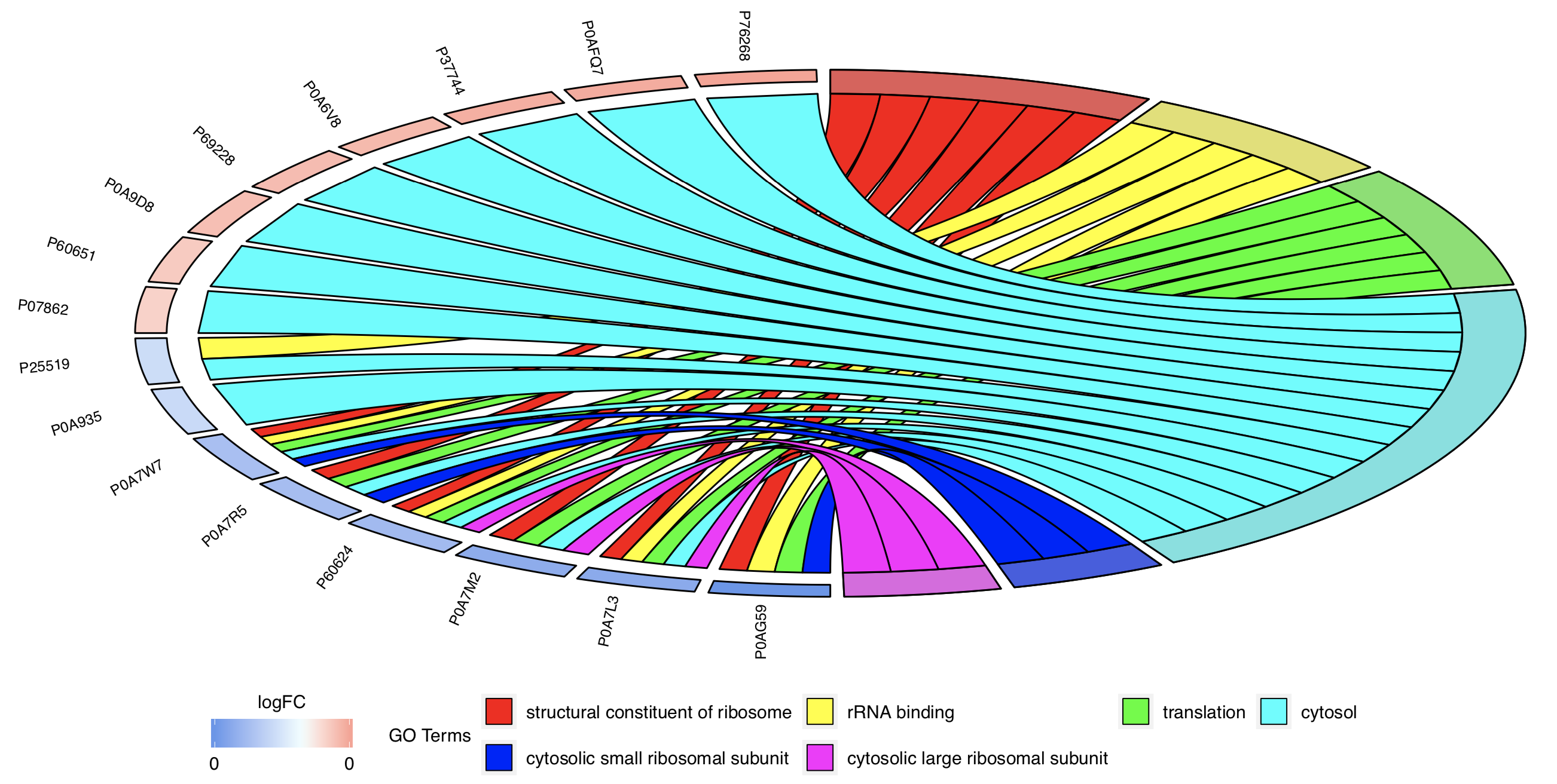


**Supplementary Figure 21. Enriched terms of differential proteins between the samples of 100% and 21% oxygen incubation.** Most of differential genes were enriched in the terms about the translation process. The protein information was shown in Table S2.


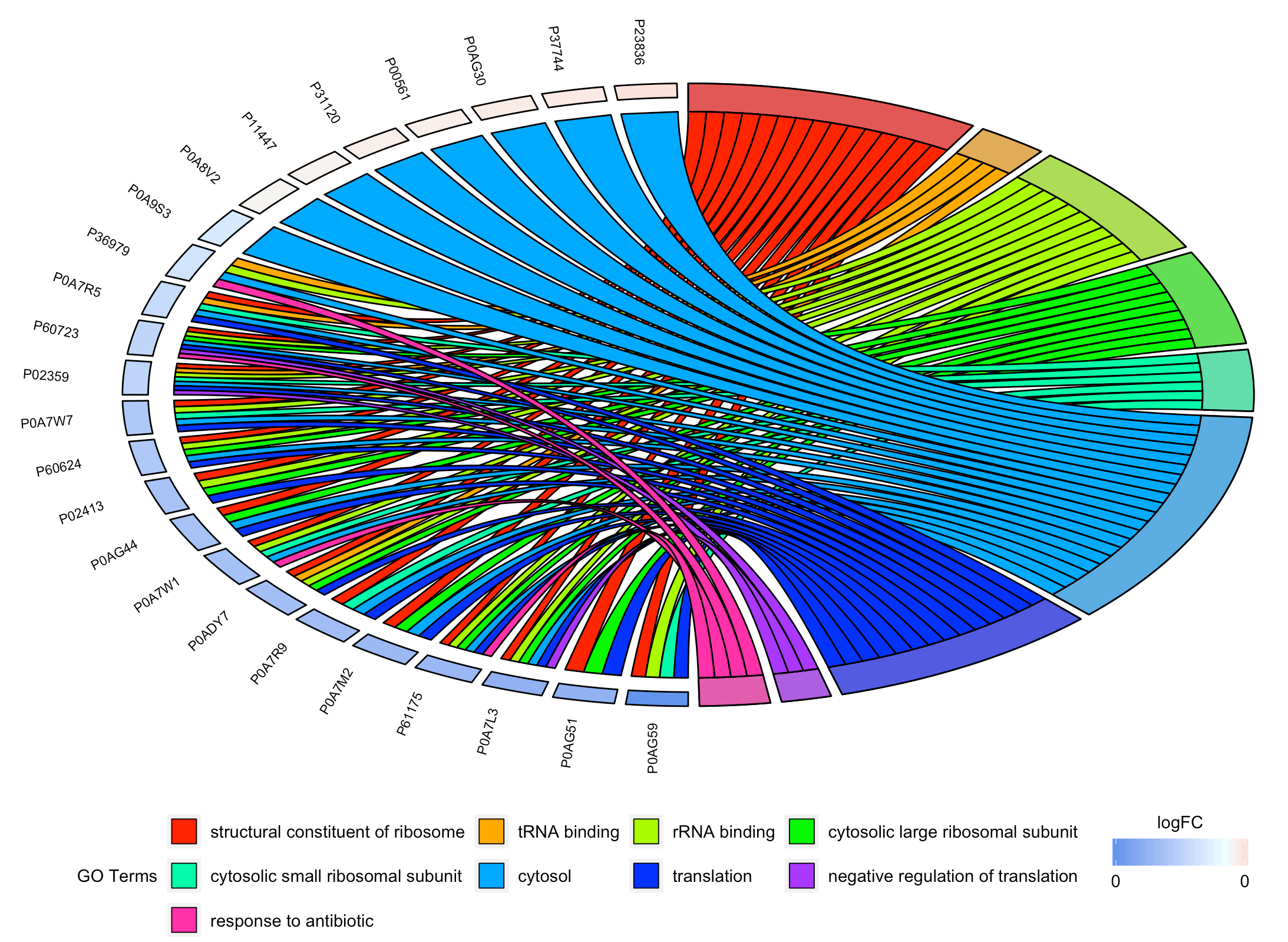


**Supplementary Figure 22. Enriched terms of differential proteins between the samples of 100% and 0% oxygen incubation.** Most of differential genes were enriched in the terms about translation process. The protein information was shown in Table S3.

**
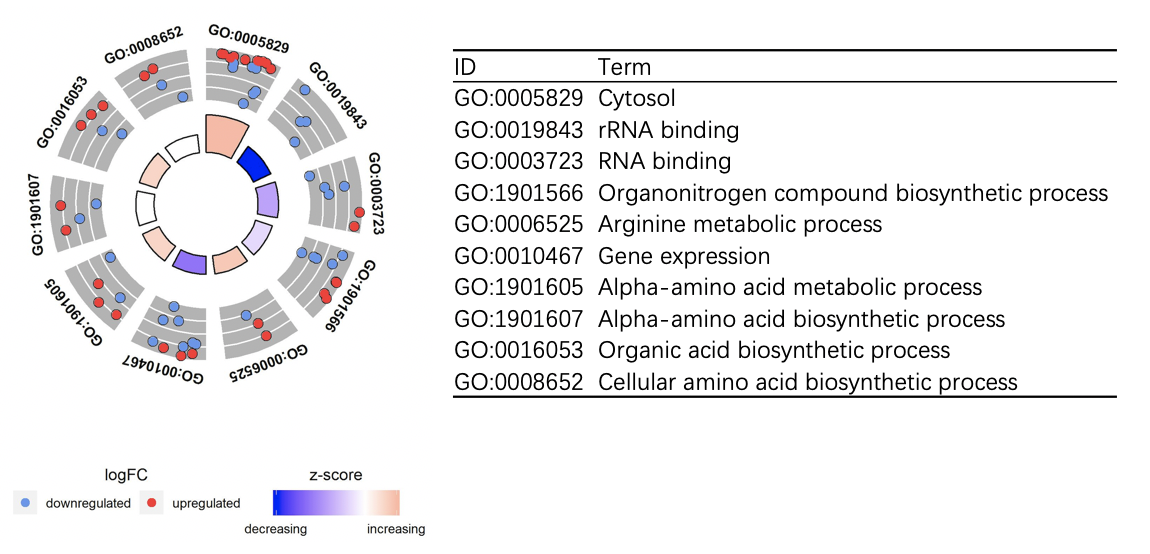
**

**Supplementary Figure 23. GOCircle and GO description of the comparison from the 21% oxygen treatments to 0% oxygen treatments.**

**
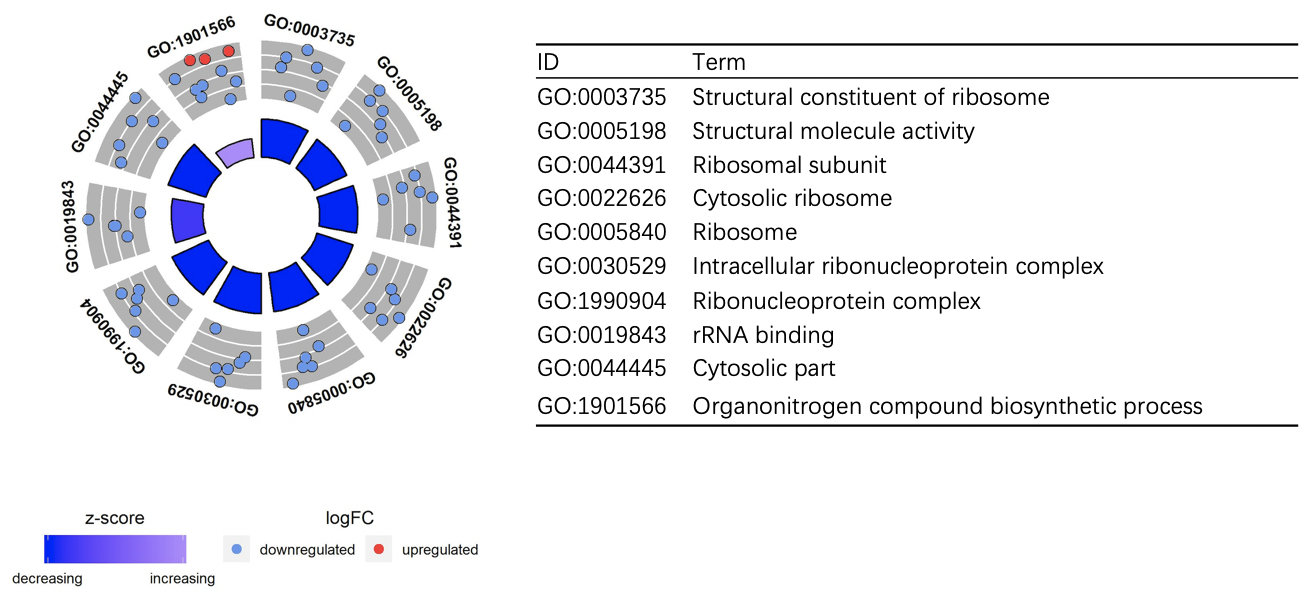
**

**Supplementary Figure 24. GOCircle and GO description of the comparison from the 100% oxygen treatments to 21% oxygen treatments.**

**
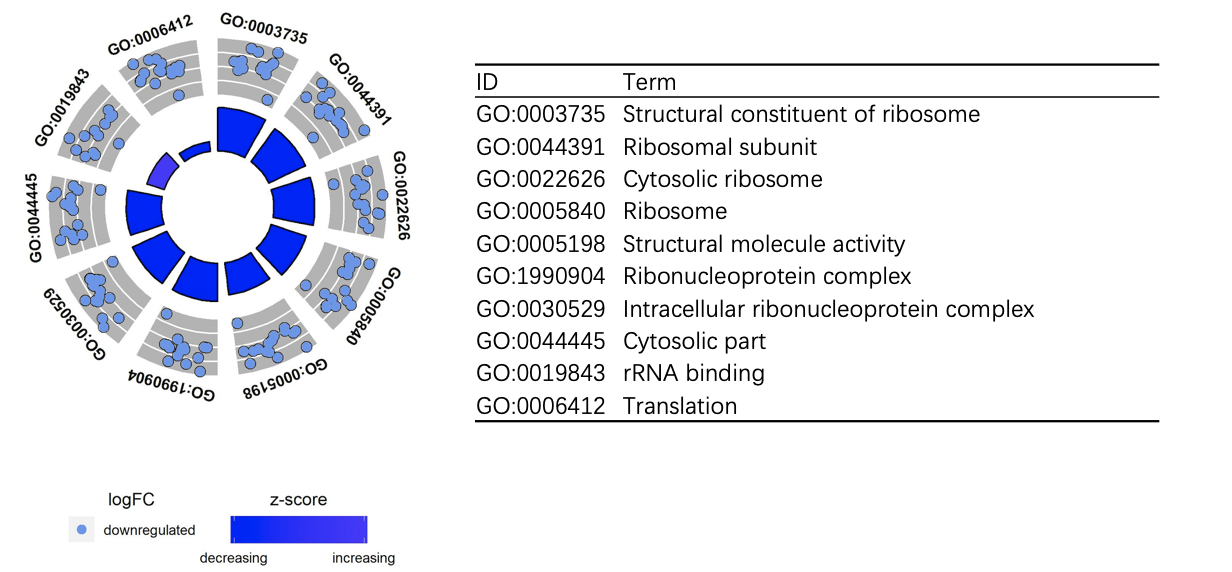
**

**Supplementary Figure 25. GOCircle and GO description of the comparison from the 100% oxygen treatments to 0% oxygen treatments.**

**Supplementary Tables**

**Table S1. The list of differential proteins in the samples treated by 21% and 0% oxygen.**

| Uniprot accession | Symbol | Description |
| --- | --- | --- |
| P0AG30 | rho | Transcription termination factor Rho |
| P0ABF1 | pcnB | Poly(A) polymeraseI |
| P00561 | thrA | Bifunctional aspartokinase/homoserinedehydrogenase1 |
| P31120 | glmM | Phosphoglucosaminemutase |
| P11447 | argH | Argininosuccinatelyase |
| P11446 | argC | N-acetyl-gamma-glutamyl-phosphatereductase |
| P45799 | nudE | ADPcompoundshydrolaseNudE |
| P37744 | rfbA | Glucose-1-phosphatethymidylyltransferase1 |
| P0ACN7 | cytR | HTH-typetranscriptionalrepressorCytR |
| P60651 | speB | Agmatinase |
| P0A9L3 | fklB | FKBP-type22kDapeptidyl-prolylcis-transisomerase |
| P0A6V8 | glk | Glucokinase |
| P0AFQ7 | ycfH | Uncharacterizedmetal-dependenthydrolaseYcfH |
| P36979 | rlmN | Dual-specificityRNAmethyltransferaseRlmN |
| P76268 | kdgR | TranscriptionalregulatorKdgR |
| P0A7S9 | rpsM | 30SribosomalproteinS13 |
| P61175 | rplV | 50SribosomalproteinL22 |
| P0AG59 | rpsN | 30SribosomalproteinS14 |

**Table S2. The list of differential proteins in the samples treated by 100% and 21% oxygen.**

| ID | Symbol | Description |
| --- | --- | --- |
| P76268 | kdgR | Transcriptional regulator KdgR |
| P0AFQ7 | ycfH | Uncharacterized metal-dependent hydrolase YcfH |
| P37744 | rfbA | Glucose-1-phosphatethymidylyl transferase 1 |
| P0A6V8 | glk | Glucokinase |
| P69228 | baeR | TranscriptionalregulatoryproteinBaeR |
| P0A9D8 | dapD | 2,3,4,5-tetrahydropyridine-2,6-dicarboxylateN-succinyltransferase |
| P60651 | speB | Agmatinase |
| P07862 | ddlB | D-alanine--D-alanineligaseB |
| P25519 | hflX | GTPaseHflX |
| P0A935 | mltA | Membrane-boundlyticmureintransglycosylaseA |
| P0A7W7 | rpsH | 30SribosomalproteinS8 |
| P0A7R5 | rpsJ | 30SribosomalproteinS10 |
| P60624 | rplX | 50SribosomalproteinL24 |
| P0A7M2 | rpmB | 50SribosomalproteinL28 |
| P0A7L3 | rplT | 50SribosomalproteinL20 |
| P0AG59 | rpsN | 30SribosomalproteinS14 |

**Table S3. The list of differential proteins in the samples treated by 100% and 0% oxygen.**

| ID | Symbol | Description |
| --- | --- | --- |
| P23836 | phoP | TranscriptionalregulatoryproteinPhoP |
| P37744 | rfbA | Glucose-1-phosphatethymidylyltransferase1 |
| P0AG30 | rho | TranscriptionterminationfactorRho |
| P00561 | thrA | Bifunctionalaspartokinase/homoserinedehydrogenase1 |
| P31120 | glmM | Phosphoglucosaminemutase |
| P11447 | argH | Argininosuccinatelyase |
| P0A8V2 | rpoB | DNA-directedRNApolymerasesubunitbeta |
| P0A9S3 | gatD | Galactitol1-phosphate5-dehydrogenase |
| P36979 | rlmN | Dual-specificityRNAmethyltransferaseRlmN |
| P0A7R5 | rpsJ | 30SribosomalproteinS10 |
| P60723 | rplD | 50SribosomalproteinL4 |
| P02359 | rpsG | 30SribosomalproteinS7 |
| P0A7W7 | rpsH | 30SribosomalproteinS8 |
| P60624 | rplX | 50SribosomalproteinL24 |
| P02413 | rplO | 50SribosomalproteinL15 |
| P0AG44 | rplQ | 50SribosomalproteinL17 |
| P0A7W1 | rpsE | 30SribosomalproteinS5 |
| P0ADY7 | rplP | 50SribosomalproteinL16 |
| P0A7R9 | rpsK | 30SribosomalproteinS11 |
| P0A7M2 | rpmB | 50SribosomalproteinL28 |
| P61175 | rplV | 50SribosomalproteinL22 |
| P0A7L3 | rplT | 50SribosomalproteinL20 |
| P0AG51 | rpmD | 50SribosomalproteinL30 |
| P0AG59 | rpsN | 30SribosomalproteinS14 |

**Table S4. The DNA sequence of sfGFP protein with 6 x His tag.**

| Gene name | Sequence |
| --- | --- |
| sfGFP | ATGCGTAAAGGCGAAGAGCTGTTCACTGGTGTCGTCCCTATTCTGGTGGAACTGGATGGTGATGTCAACGGTCATAAGTTTTCCGTGCGTGGCGAGGGTGAAGGTGACGCAACTAATGGTAAACTGACGCTGAAGTTCATCTGTACTACTGGTAAACTGCCGGTACCTTGGCCGACTCTGGTAACGACGCTGACTTATGGTGTTCAGTGCTTTGCTCGTTATCCGGACCATATGAAGCAGCATGACTTCTTCAAGTCCGCCATGCCGGAAGGCTATGTGCAGGAACGCACGATTTCCTTTAAGGATGACGGCACGTACAAAACGCGTGCGGAAGTGAAATTTGAAGGCGATACCCTGGTAAACCGCATTGAGCTGAAAGGCATTGACTTTAAAGAAGACGGCAATATCCTGGGCCATAAGCTGGAATACAATTTTAACAGCCACAATGTTTACATCACCGCCGATAAACAAAAAAATGGCATTAAAGCGAATTTTAAAATTCGCCACAACGTGGAGGATGGCAGCGTGCAGCTGGCTGATCACTACCAGCAAAACACTCCAATCGGTGATGGTCCTGTTCTGCTGCCAGACAATCACTATCTGAGCACGCAAAGCGTTCTGTCTAAAGATCCGAACGAGAAACGCGATCATATGGTTCTGCTGGAGTTCGTAACCGCAGCGGGCATCACGCATGGTATGGATGAACTGTACAAACATCACCATCACCATCATTAA |
